# Uncoupling protein 2 and aldolase B impact insulin release by modulating mitochondrial function and Ca^2+^ release from the ER

**DOI:** 10.1016/j.isci.2022.104603

**Published:** 2022-06-14

**Authors:** Ryota Inoue, Takahiro Tsuno, Yu Togashi, Tomoko Okuyama, Aoi Sato, Kuniyuki Nishiyama, Mayu Kyohara, Jinghe Li, Setsuko Fukushima, Tatsuya Kin, Daisuke Miyashita, Yusuke Shiba, Yoshitoshi Atobe, Hiroshi Kiyonari, Kana Bando, A.M. James Shapiro, Kengo Funakoshi, Rohit N. Kulkarni, Yasuo Terauchi, Jun Shirakawa

**Affiliations:** 1 Laboratory of Diabetes and Metabolic Disorders, Institute for Molecular and Cellular Regulation (IMCR), Gunma University, 3-39-15 Showa-machi, Maebashi 371-8512, Japan; 2 Department of Endocrinology and Metabolism, Graduate School of Medicine, Yokohama City University, Yokohama 236-0004, Japan; 3 Clinical Islet Laboratory and Clinical Islet Transplant Program, University of Alberta, Edmonton, AB T6G2C8, Canada; 4 Department of Neuroanatomy, Yokohama City University School of Medicine, Yokohama 236-0004, Japan; 5 Laboratory for Animal Resources and Genetic Engineering, RIKEN Center for Biosystems Dynamics Research, Kobe 650-0047, Japan; 6 Islet Cell and Regenerative Biology, Joslin Diabetes Center, Department of Medicine, Beth Israel Deaconess Medical Center, Harvard Stem Cell Institute, Harvard Medical School, Boston, MA 02215, USA

**Keywords:** Endocrinology, cell biology, Functional aspects of cell biology

## Abstract

Uncoupling protein 2 (UCP2), a mitochondrial protein, is known to be upregulated in pancreatic islets of patients with type 2 diabetes (T2DM); however, the pathological significance of this increase in UCP2 expression is unclear. In this study, we highlight the molecular link between the increase in UCP2 expression in β-cells and β-cell failure by using genetically engineered mice and human islets. β-cell-specific UCP2-overexpressing transgenic mice (βUCP2Tg) exhibited glucose intolerance and a reduction in insulin secretion. Decreased mitochondrial function and increased aldolase B (AldB) expression through oxidative-stress-mediated pathway were observed in βUCP2Tg islets. AldB, a glycolytic enzyme, was associated with reduced insulin secretion via mitochondrial dysfunction and impaired calcium release from the endoplasmic reticulum (ER). Taken together, our findings provide a new mechanism of β-cell dysfunction by UCP2 and AldB. Targeting the UCP2/AldB axis is a promising approach for the recovery of β-cell function.

## Highlights

• Increased UCP2 expression impairs β-cell function independent of proton leak
• UCP2 induces the expression of AldB, a glycolytic enzyme, in β-cells
• Mitochondrial function and Ca^2+^ release from the ER are dysregulated by AldB
• Knockdown of AldB rescues UCP2-mediated β-cell failure

## Graphical abstract

**Figure:**
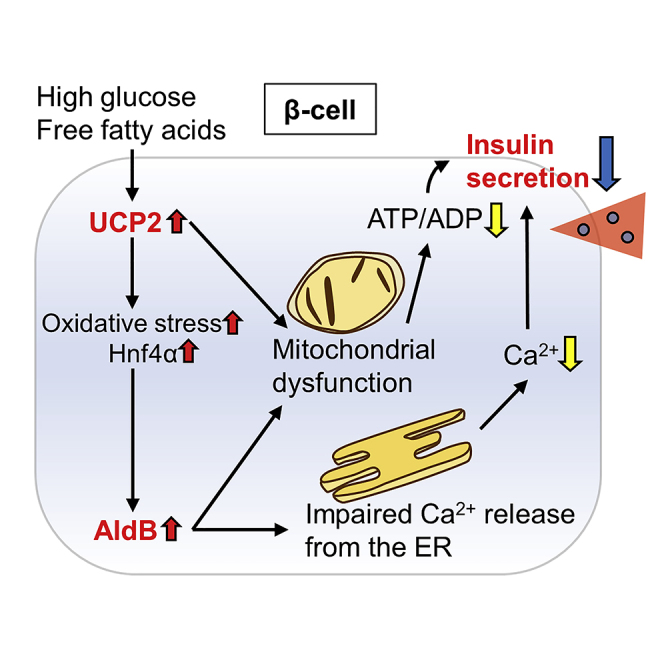

## Introduction

Type 2 diabetes mellitus (T2DM) occurs as a consequence of β-cell dysfunction in a background of systemic insulin resistance (Kahn, 2000). Insulin secretion from pancreatic β-cells is tightly regulated to maintain blood glucose levels within a narrow range. Mitochondrial oxidative phosphorylation (OXPHOS) is the predominant source of ATP in β-cells, and patients with mtDNA mutations exhibit β-cell dysfunction and overt diabetes due to reduced ATP production (Erecińska et al., 1992; Gerbitz et al., 1996).

Uncoupling proteins (UCPs) create proton leaks in the mitochondrial inner membrane, which dissipate the proton motive force, thereby bypassing energy conservation by ATP synthase, and the energy is dissipated as heat. UCP1 is specifically expressed in brown adipocytes and is known to play a role in thermogenesis (Nedergaard et al., 2001). Moreover, UCP2, which has 59% amino acid identity to UCP1 in humans, is expressed widely in metabolic tissues, including pancreatic β-cells (Fleury et al., 1997). UCP2 protein levels have been reported to be increased in the islets of individuals with T2DM compared with those from nondiabetic humans (Anello et al., 2005). It remains unclear how UCP2 affects the development of diabetes, yet some research groups have used either UCP2-overexpressing mouse islets or β-cell-specific UCP2-knockout mice (Chan et al., 1999; Produit-Zengaffinen et al., 2007; Robson-Doucette et al., 2011; Zhang et al., 2001). The possibility that UCP2 does not act as an uncoupler has also been suggested (Galetti et al., 2009; Nicholls, 2021).

To clarify the significance of UCP2 expression in pancreatic β-cells in relation to the pathogenesis of T2DM, we investigated the regulatory mechanisms underlying UCP2 expression in β-cells and analyzed the phenotype of mice with a β-cell-specific UCP2-overexpressing transgene (βUCP2Tg). We also focused on the role of aldolase B (AldB) in β-cells, which was the most upregulated gene in islets from βUCP2Tg mice.

## Results

### UCP2 expression is increased in the islets of mice with T2DM

**Figure 1:**
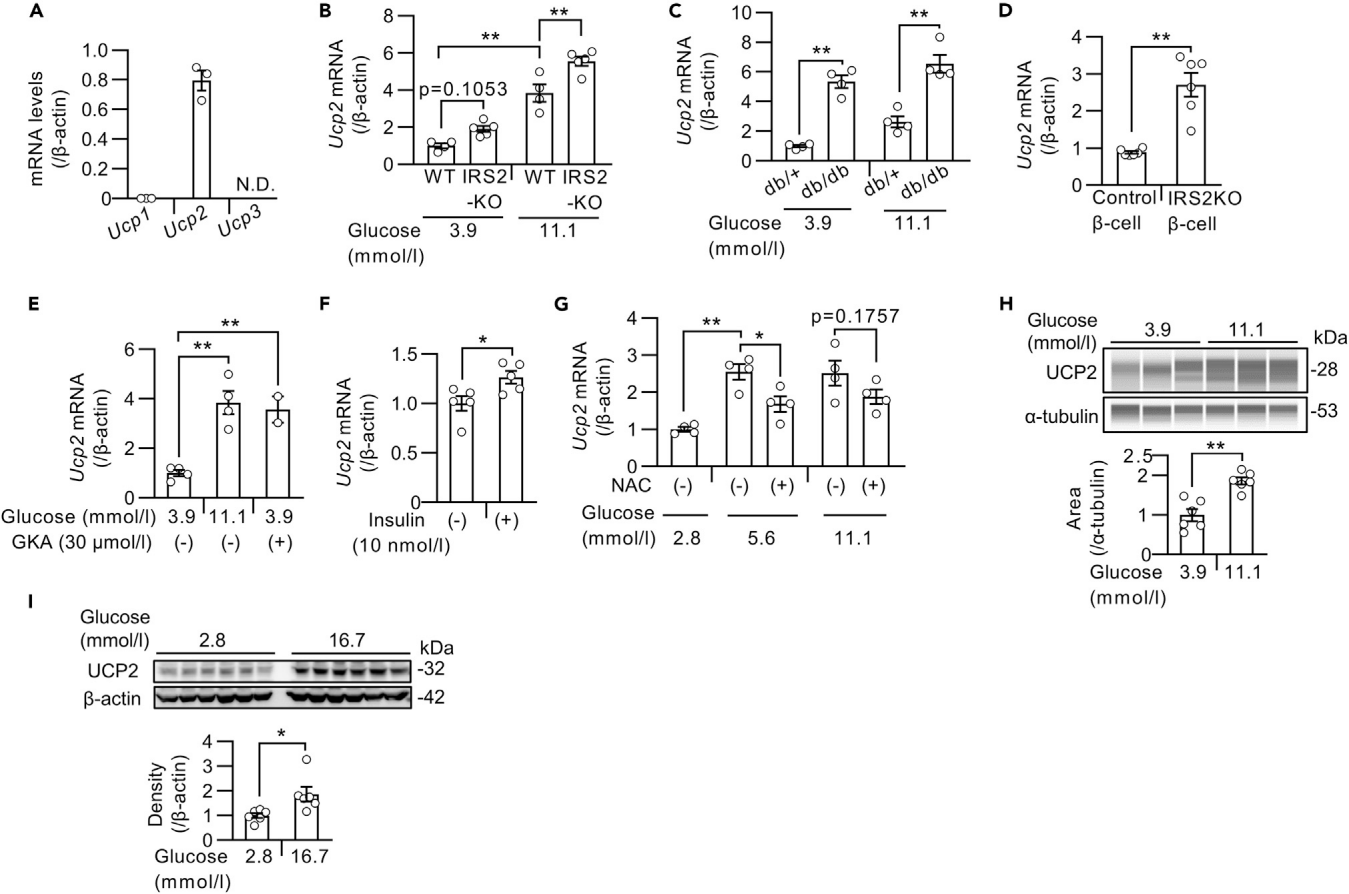
UCP2 expression was increased in islets exposed to type 2 diabetes-like conditions (A) mRNA levels of *Ucp1*, *Ucp2*, and *Ucp3* in islets from C57BL/6J mice (n = 3). N.D.: not detected. (B–G) *Ucp2* mRNA levels in mouse islets and β-cell lines. (B) Islets from IRS2KO and WT mice were incubated with 3.9 or 11.1 mmol/L glucose for 24 h (WT: n = 4, IRS2KO: n = 5). (C) Islets from db/db and db/+ mice were incubated with 3.9 or 11.1 mmol/L glucose for 24 h (n = 4 per group). (D) IRS2KO β-cell lines and control β-cell lines were incubated with 25 mmol/L glucose for 24 h (n = 6 per group). (E) Islets from C57BL/6J mice were incubated with 3.9 or 11.1 mmol/L glucose with or without 30 μmol/L GKA for 24 h (low- and high-glucose group: n = 4, GKA group: n = 2). (F) Starved islets from C57BL/6J mice were treated with or without 10 nmol/L insulin for 12 h (n = 5 per group). (G) Islets from C57BL/6J mice were incubated with 2.8, 5.6, or 11.1 mmol/L glucose with or without 20 mmol/L NAC for 24 h (n = 4 per group). (H) Western blots of UCP2 and α-tubulin in islets from C57BL/6J mice. Islets were incubated with 3.9 or 11.1 mmol/L glucose for 24 h (n = 6 per group). Western blotting was performed by Abby system. (I) Western blots of UCP2 and α-tubulin in INS1 832/13 cells. Cells were incubated with 2.8 or 16.7 mmol/L glucose for 24 h (n = 6 per group). Data are the means ± SEM ∗p < 0.05, ∗∗p < 0.01. The two-tailed Student’s t test was used in (D), (F), (H), and (I). One-way ANOVA was used in (B), (C), (E), and (G).

First, we measured the expression levels of *Ucp2* in mouse islets. Among the UCP subtypes, *Ucp2*, rather than *Ucp1* or *Ucp3*, was predominantly expressed in mouse islets (Figure 1A). Because UCP2 was reported to be increased in islets from T2DM donors (Anello et al., 2005), we measured *Ucp2* expression in islets from insulin receptor substrate (Irs)-2-deficient (IRS2KO) mice (Kubota et al., 2000) and BKS.Cg-Dock7m+/+Leprdb/J (db/db) mice, which are models of T2DM that exhibit both β-cell dysfunction and insulin resistance. An increase in *Ucp2* expression in islets from both IRS2KO and db/db mice was observed compared with their corresponding controls (Figures 1B and 1C). To assess whether *Ucp2* expression is induced by high glucose, we also cultured islets from diabetic mice at different glucose levels, and a further increase in *Ucp2* expression was observed in the presence of high glucose levels (Figures 1B and 1C). Upregulation of *Ucp2* was also observed in IRS2KO β-cell lines, which were generated as previously described (Assmann et al., 2009; Kulkarni et al., 1999), suggesting that it was secondary to β-cell dysfunction (Figure 1D). To evaluate the effect of glucose signaling on changes in *Ucp2* expression, we treated the islets with glucose, a glucokinase activator (GKA) (Figure 1E), or insulin (Figure 1F), and in all conditions, the expression of *Ucp2* was increased. Moreover, the upregulation of *Ucp2* induced by high glucose concentration was partially inhibited by N-acetylcysteine (NAC), an antioxidant (Figure 1G). We also confirmed the increased protein levels of UCP2 in mouse islets and INS1 β-cells in the presence of high glucose concentrations (Figures 1H and 1I).

Because the activation of glucose signaling by GKA inhibited the apoptosis induced by ER stress (Shirakawa et al., 2013), we examined the effect of ER stress on *Ucp2* expression in mouse islets. The expression of *Ucp2* in mouse islets was not changed by treatment with thapsigargin, an inducer of ER stress (Figure S1A). We next assessed the effects of insulin receptor signaling on *Ucp2* expression. OSI-906, a dual inhibitor of IGF-1 and insulin receptors, had no effect on *Ucp2* expression in mouse islets (Figure S1B). Conversely, islet *Ucp2* expression was decreased by treatment with Akt inhibitor X, a selective inhibitor of Akt phosphorylation (Figure S1C), or U0126, an ERK inhibitor (Figure S1D). We also explored the effect of potassium channel activity and calcium signaling on *Ucp2* expression, which are important in glucose-stimulated insulin secretion, and no changes in *Ucp2* expression were observed in mouse islets treated with diazoxide, a potassium channel opener (Figure S1E); nifedipine, an L-type calcium channel blocker; or FK506, a calcineurin inhibitor (Figure S1F). The mammalian target of rapamycin (mTOR) signal is activated by insulin signaling in pancreatic β-cells, but the mTOR inhibitor rapamycin had no effect on islet *Ucp2* expression under high glucose conditions (Figure S1G).

These results indicated that the expression of *Ucp2* was upregulated via Akt or ERK pathways under diabetes-like pathophysiological conditions, such as hyperglycemia and oxidative stress.

### Overexpression of UCP2 in β-cells impairs insulin secretion

**Figure 2:**
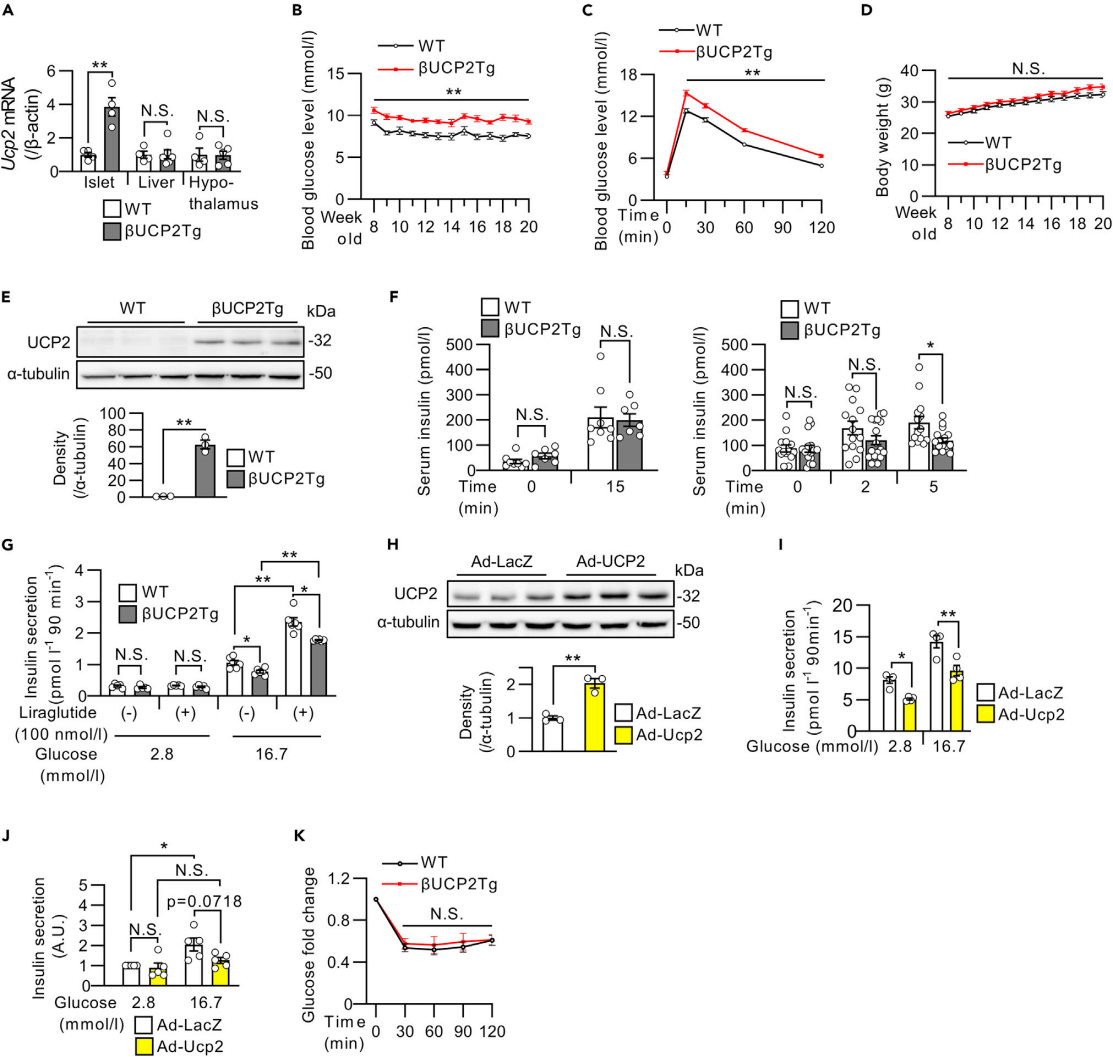
βUCP2Tg mice showed impaired glucose tolerance with reduced insulin secretion (A) *Ucp2* mRNA levels in the islets, livers, and hypothalamus from βUCP2Tg mice and their littermate WT mice (WT: n = 4, βUCP2Tg: n = 5–6). (B) Blood glucose levels in βUCP2Tg and WT mice (WT: n = 13, βUCP2Tg: n = 21). (C) OGTT in βUCP2Tg and WT mice (WT: n = 29, βUCP2Tg n = 36, 1.5 g/kg BW glucose). (D) Body weight changes in βUCP2Tg and WT mice (WT: n = 13, βUCP2Tg: n = 21). (E) Western blot of UCP2 and α-tubulin in βUCP2Tg and WT islets. Islets were incubated with 11.1 mmol/L glucose for 24 h (n = 3 per group). (F) Serum insulin levels during the OGTT in βUCP2Tg and WT mice at 0 and 15 min (left panel, WT: n = 7, βUCP2Tg: n = 8, 1.5 g/kg BW glucose) and at 0, 2, and 5 min (right panel, WT: n = 14, βUCP2Tg: n = 17, 2.5 g/kg BW glucose). (G) Glucose-stimulated insulin secretion (GSIS) in βUCP2Tg and WT islets. Islets were incubated in KRB buffer containing 2.8 or 16.7 mmol/L glucose for 90 min with or without 100 nmol/L liraglutide (n = 5 per group). (H) Western blot of UCP2 and α-tubulin in MIN6-M9 cells. Cells were infected with Ad-LacZ or Ad-Ucp2 at an MOI of 500 for 48 h (n = 3 per group). (I) GSIS in MIN6-M9 cells. Cells were infected with Ad-LacZ or Ad-Ucp2 at an MOI of 500 for 48 h and then incubated with 2.8 or 16.7 mmol/L glucose for 90 min (n = 4 per group). (J) GSIS in human islets. Islets were infected with Ad-LacZ or Ad-Ucp2 at 3 × 10^6^ MOI and cultured at 5.6 mmol/L glucose for 48 h. Then, islets were incubated in KRB buffer containing 2.8 or 16.7 mmol/L glucose for 60 min (n = 5 per group). Insulin concentration was normalized by insulin content in islets. The presented data are the ratio to the value from islets infected with Ad-LacZ control for each donor. (K) Fold-changes in blood glucose concentrations in βUCP2Tg and WT mice in the insulin tolerance test (ITT) (WT: n = 15, βUCP2Tg: n = 12, 0.75 U/kg BW insulin). Data are the means ± SEM ∗p < 0.05, ∗∗p < 0.01. N.S.: not significant. The two-tailed Student’s t test was used in (A-F), (H), and (K). One-way ANOVA was used in (G), (I), and (J).

To investigate the impact of UCP2 upregulation in diabetic β-cells, we generated βUCP2Tg mice. These mice overexpress the full-length *Ucp2* gene specifically in pancreatic β-cells under rat insulin promoter activity (Figure S2A). βUCP2Tg mice showed significantly increased *Ucp2* expression in islets but not in livers or hypothalamus compared with the corresponding wild-type (WT) mice (Figure 2A). βUCP2Tg mice exhibited significantly impaired glucose tolerance (Figures 2B and 2C). No changes were observed in body weights in βUCP2Tg mice compared with the controls (Figure 2D). We then validated the increased protein levels of UCP2 in islets from βUCP2Tg mice (βUCP2Tg islets) (Figure 2E). Moreover, there was no change in the expression level of *Ucp1* in βUCP2Tg islets (Figure S2B), suggesting an absence of compensatory effect between the isoforms. Serum insulin levels were significantly reduced in βUCP2Tg mice at 5 but not 15 min after glucose loading (Figure 2F). The insulinogenic index was decreased in βUCP2Tg mice at 2 and 5 min after glucose loading (Figure S2C). The βUCP2Tg islets showed decreased insulin secretion in response to glucose stimulation (Figure 2G). Conversely, liraglutide, a GLP-1 receptor agonist, enhanced insulin secretion in both mice (Figure 2G). Furthermore, adenovirus-vector-induced expression of *Ucp2* reduced insulin secretion in MIN6-M9 cells and human islets (Figures 2H–2J and S2D). The insulin sensitivity in βUCP2Tg mice was similar to that in WT mice (Figure 2K). β-cell mass and β-cell proliferative activity in βUCP2Tg mice were comparable to those in WT animals (Figures S2E and S2F).

### βUCP2Tg islets exhibited abnormalities in mitochondrial morphology and function

**Figure 3:**
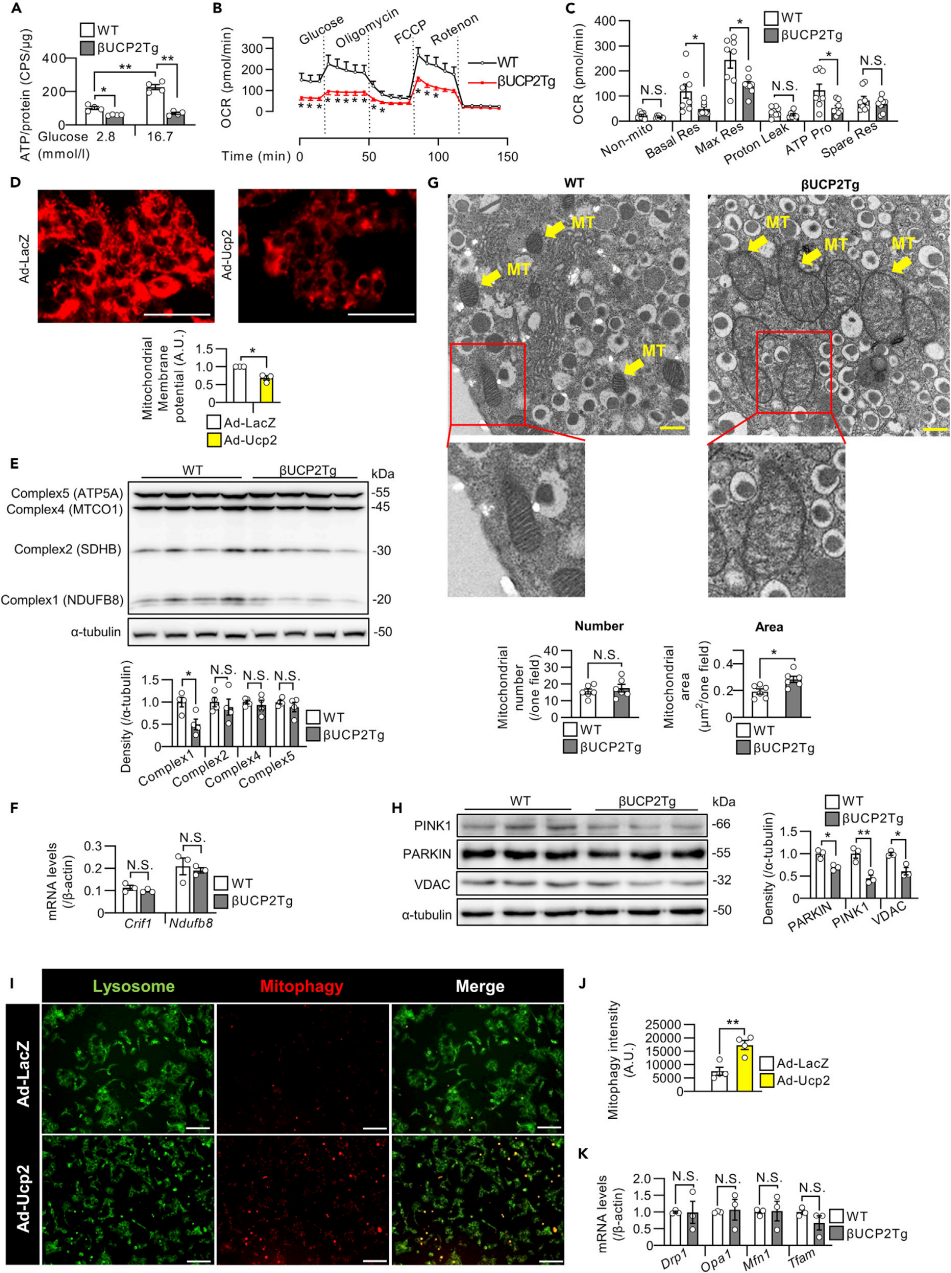
UCP2-overexpressing β-cells exhibited abnormalities in mitochondrial morphology and function (A) ATP levels in βUCP2Tg and WT islets. Ten isolated islets were incubated with 2.8 mmol/L glucose for 30 min and then stimulated with 2.8 or 16.7 mmol/L glucose for 10 min (n = 4 per group). (B and C) Oxygen consumption rate in βUCP2Tg and WT islets (n = 8 per group). The graph shows nonmitochondrial oxygen consumption, basal respiration, maximal respiration, proton leakage, ATP production, and spare respiratory capacity. (D) Mitochondrial membrane potential of MIN6-M9 cells. The graph shows the intensities of MT-1 dye in MIN6-M9 cells. The scale bar indicates 100 μm. (E) Western blot of mitochondrial OXPHOS proteins and α-tubulin in βUCP2Tg and WT islets. Islets were incubated with 11.1 mmol/L glucose for 24 h (n = 4 per group). (F) mRNA levels of *Crif1* and *Ndufb8* in βUCP2Tg and WT islets. Islets were incubated with 11.1 mmol/L glucose for 24 h (n = 3 per group). (G) Morphology of β-cells under an electron microscope. The scale bar indicates 500 nm. The number of mitochondria (left panel) and the percent area of the mitochondria (right panel) in β-cells (n = 6 per group). The mitochondrial number and area per field were calculated using ImageJ software. MT; mitochondria. (H) Western blot of mitophagy-associated proteins and α-tubulin in βUCP2Tg and WT islets. Islets were incubated with 11.1 mmol/L glucose for 24 h (n = 3 per group). (I and J) The fluorescence of MtPhagy dye and Lyso dye in live MIN6-M9 cells. Cells were transfected with Ad-LacZ or Ad-Ucp2 at an MOI of 500 for 48 h. The images are representative of four independent experiments. The scale bar indicates 100 μm. (J) The intensity of MtPhagy dye was calculated using ImageJ software (n = 4 per group). (K) mRNA levels of *Drp1*, *Opa1*, *Mfn1*, and *Tfam* in βUCP2Tg and WT islets. Islets were incubated with 5.6 mmol/L glucose for 24 h (n = 3 per group). Data are the means ± SEM ∗p < 0.05, ∗∗p < 0.01. N.S.: not significant. The two-tailed Student’s t test was used in (B-H), (J), and (K). One-way ANOVA was used in (A).

Because UCP2 localizes to the inner mitochondrial membrane, we investigated mitochondrial function in βUCP2Tg islets. The βUCP2Tg islets showed a significant decrease in ATP production in response to glucose stimulation (Figure 3A). Analysis of mitochondrial respiration revealed that mitochondrial basal respiration, maximal respiration, and ATP production were all significantly lower, whereas no change in proton leakage was noted in βUCP2Tg islets (Figures 3B and 3C). Furthermore, adenovirus-vector-induced overexpression of *Ucp2* reduced mitochondrial membrane potential in MIN6-M9 cells, suggesting that mitochondrial dysfunction was induced by UCP2 (Figure 3D). We found that the protein level of NDUFB8, a component of mitochondrial OXPHOS complex 1, was also significantly decreased in βUCP2Tg islets (Figure 3E). Meanwhile, there were no significant differences in the mRNA expression of *Ndufb8*, a complex 1 gene, and *Crif1*, which is associated with the synthesis of mtDNA-encoded OXPHOS polypeptides between βUCP2Tg and WT islets (Figure 3F). Dilatation of mitochondria with loss of cristae was observed in βUCP2Tg β-cells by electron microscopy (Figure 3G).

Mitophagy, which refers to selective mitochondrial autophagy, is critical for the removal of damaged mitochondria (Lemasters et al., 1998). In βUCP2Tg islets, the protein expression levels of PARKIN and PINK1, which are known to be mitophagy-related proteins (Narendra et al., 2008), were significantly decreased (Figure 3H). It has been reported that upregulated mitophagy flux reduces the expression of PARKIN due to increased protein turnover in the MIN6 β-cell line (Sidarala et al., 2020). We measured the mitophagy flux in UCP2-overexpressing MIN6-M9 cells, and the mitophagy flux was significantly increased by UCP2 (Figures 3I and 3J). These results indicated that the mitochondrial dysfunction induced by UCP2 enhanced mitophagy, resulting in decreased mitophagy-related protein expression. Moreover, the expression levels of *Drp1*, *Opa1*, and *Mfn1*, which are related to mitochondrial fission and fusion (Zorzano et al., 2010), and *Tfam*, a mitochondrial transcription factor (Ekstrand et al., 2004), remained unchanged in βUCP2Tg islets (Figure 3K).

### Aldolase B is induced by UCP2 in pancreatic β-cells

**Figure 4:**
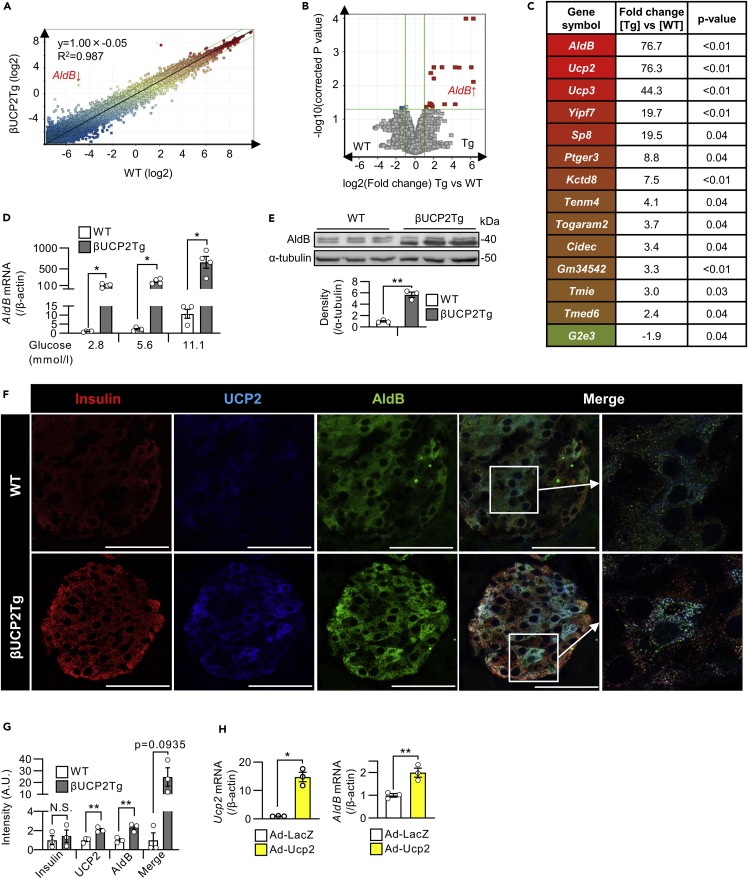
Aldolase B was increased in βUCP2Tg β-cells (A–C) DNA microarray in βUCP2Tg and WT islets (n = 4 per group). Islets were incubated with 11.1 mmol/L glucose for 8 h. (A) Scatterplot. (B) Volcano plot. (C) Heatmap. The graph shows the gene symbols, fold-change (βUCP2Tg versus WT), and p value. (D) Aldolase B (*AldB*) mRNA levels in islets from βUCP2Tg and WT mice. Islets were incubated with 2.8, 5.6, or 11.1 mmol/L glucose for 24 h (n = 4). (E) Western blot of AldB and α-tubulin in βUCP2Tg and WT islets. Islets were incubated with 11.1 mmol/L glucose for 24 h (n = 3 per group). (F and G) Immunostaining of βUCP2Tg and WT islets. Insulin is stained red, UCP2 is stained blue, and AldB is stained green. The scale bar represents 50 μm. (G) The intensity of insulin, UCP2, and AldB staining was calculated using ImageJ software (n = 3 per group). The intensity in the overlapping region of UCP2 and AldB staining is also represented as a merge. (H) *Ucp2* (left panel) and *AldB* (right panel) mRNA levels in C57BL/6J islets. Islets infected with Ad-LacZ or Ad-Ucp2 (3 × 10^6^ MOI) were incubated with 5.6 mmol/L glucose for 24 h (n = 3 per group). Data are presented as the means ± SEM ∗p < 0.05, ∗∗p < 0.01. N.S.: not significant. The two-tailed Student’s t test was used in (D), (E), (G), and (H).

To identify the target genes of UCP2 in pancreatic β-cells, we performed gene expression microarray analysis in βUCP2Tg islets. We identified 13 upregulated genes (>1.5-fold, p < 0.05) and one downregulated gene (<0.67-fold, p < 0.05) in βUCP2Tg islets and validated the mRNA levels by qPCR (Figures 4A–4C and S3A). The most upregulated gene in βUCP2Tg islets was aldolase B (*AldB*) (Figures 4C and 4D), the expression of which has been reported to be negatively associated with insulin secretion in human islets (Gerst et al., 2018). The protein levels of AldB were also increased in βUCP2Tg islets (Figure 4E). Among the aldolase subtypes, the expression of aldolase A (*AldA*) was the highest in the WT islets, whereas the βUCP2Tg islets showed higher expression levels of *AldB* than *AldA* (Figure S3B). Immunofluorescence for both UCP2 and AldB was mainly localized in β-cells, and the signal intensities for these proteins were increased in βUCP2Tg islets (Figures 4F and 4G). Stimulated emission depletion (STED) microscopy also showed increased expression of UCP2 and AldB in βUCP2Tg β-cells (Figure S3C). We confirmed that adenovirus-vector-induced expression of *Ucp2* in mouse islets increased *AldB* expression (Figure 4H). The expression of UCP2 and AldB was also detected in human β-cells (Figure S3D).

### AldB expression was regulated by high glucose and free fatty acids in β-cells

**Figure 5:**
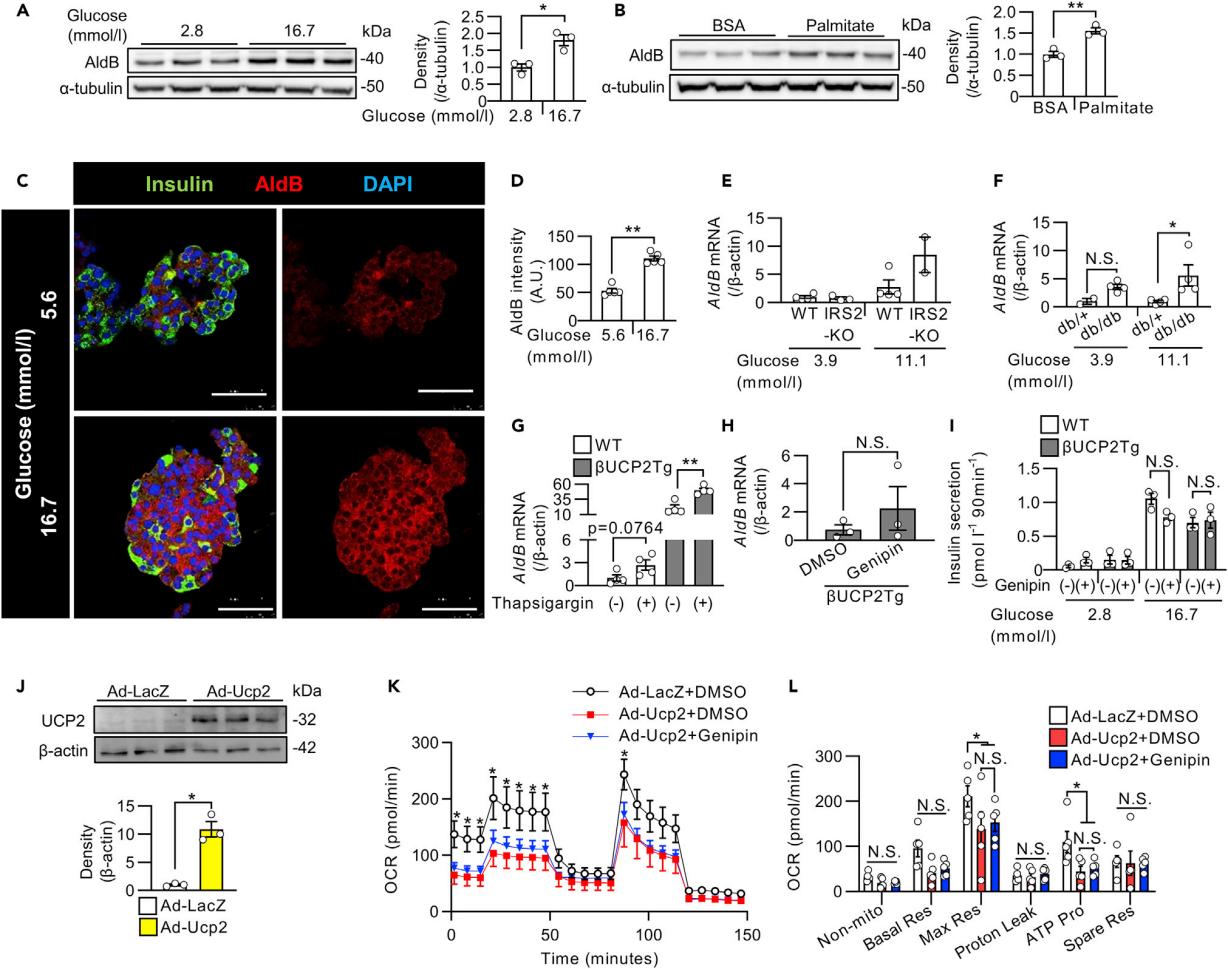
The regulation of AldB expression in islets and the effect of proton leakage by UCP2 on insulin secretion (A and B) Western blot of AldB and α-tubulin in INS1 β-cells. (A) Cells were incubated with 2.8 or 16.7 mmol/L glucose for 24 h (n = 3 per group). (B) Cells were treated with BSA or 0.5 mmol/L palmitate for 24 h (n = 3 per group). (C and D) Immunostaining of human islets. Islets were incubated with 5.6 or 16.7 mmol/L glucose for 48 h. Insulin is stained green, AldB is stained red, and DAPI is stained blue. The scale bar represents 50 μm. (D) Graph shows the intensity of AldB staining in insulin-positive β-cells (n = 5 islets from one donor). (E and F) *AldB* mRNA levels in islets from IRS2KO and WT mice (E, WT: n = 4, IRS2KO: n = 2–4) and db/db and db/+ mice (F, db/+: n = two to four, db/db: n = 4). Islets were incubated with 3.9 or 11.1 mmol/L glucose for 24 h. (G) *AldB* mRNA levels in islets from WT and βUCP2Tg mice. Islets were incubated with 5.6 mmol/L glucose with or without 1 μmol/L thapsigargin for 24 h (n = 4 per group). (H) *AldB* mRNA levels in islets from βUCP2Tg mice. Islets were incubated with 11.1 mmol/L glucose with or without 5 μmol/L genipin for 24 h (n = 3 per group). (I) GSIS in islets from WT and βUCP2Tg mice. Islets were incubated in KRB buffer containing 2.8 and 16.7 mmol/L glucose for 90 min with or without 5 μmol/L genipin (n = 3 per group). (J) Western blot of UCP2 and β-actin in mouse islets. Islets were infected with Ad-LacZ or Ad-Ucp2 at an MOI of 1 × 10^4^ and cultured in the RPMI1640 medium containing 5.6 mmol/L glucose for 24 h. (K and L) Oxygen consumption rate in islets from C57BL/6J mice. Islets were infected with Ad-LacZ or Ad-Ucp2 at an MOI of 1 × 10^4^ and cultured in the RPMI1640 medium containing 5.6 mmol/L glucose in the presence or absence of 5 μmol/L genipin for 24 h. (K) The asterisk indicates a significant difference in comparison with the Ad-Ucp2 + DMSO group. (L) The graph shows nonmitochondrial oxygen consumption, basal respiration, maximal respiration, proton leakage, ATP production, and spare respiratory capacity. Data are the means ± SEM ∗p < 0.05, ∗∗p < 0.01. N.S.: not significant. The two-tailed Student’s t test was used in (A), (B), (D), (H), and (J) One-way ANOVA was used in (E–G), (I), (K), and (L).

Next, we investigated the regulatory mechanisms of AldB expression in β-cells. The protein level of AldB in INS1 β-cells was increased by stimulation with glucose (Figure 5A) or palmitate (Figure 5B). Fluorescent immunostaining showed that the signal intensities for AldB were increased in human β-cells by high glucose concentrations (Figures 5C and 5D). Consistently, the islets from hyperglycemic IRS2KO mice (Figure 5E) and db/db mice (Figure 5F) showed a tendency toward increased *AldB* expression, similar to the case for *Ucp2* expression. Treatment with insulin, however, did not affect the expression of *AldB* in mouse islets (Figure S4A). Modulation of ER stress by treatment with thapsigargin (Figure 5G) increased the expression of *AldB* in mouse islets, unlike the case for UCP2, suggesting that AldB expression could also be induced by cellular stressors. As shown in a previous report (Ma et al., 2007), diazoxide increased *AldB* expression in mouse islets (Figure S4B). Meanwhile, neither inhibition of L-type calcium channels by nifedipine nor inhibition of calcineurin by FK-506 affected *AldB* expression (Figure S4C). Regarding insulin receptor signaling, treatment with OSI-906, an IR/IGF1R dual inhibitor, decreased *AldB* expression, whereas treatment with an Akt inhibitor increased *AldB* expression in the presence of GKA (Figures S4D and S4E). *In vitro*, OSI-906 did not inhibit insulin signaling in β-cells (Shirakawa et al., 2014), which could explain the difference in *AldB* expression between OSI-906 and Akt inhibitor.

### UCP2-mediated proton leakage did not affect insulin secretion in βUCP2Tg islets

Genipin reportedly inhibited UCP2-mediated proton leakage in mouse islets (Zhang et al., 2006). The expression of *AldB* was not suppressed by genipin in βUCP2Tg islets (Figure 5H). Therefore, we evaluated the contribution of proton leakage induced by UCP2 to the impairment of insulin secretion in βUCP2Tg β-cells by using genipin. Because genipin did not affect GSIS in either WT or βUCP2Tg islets (Figure 5I), uncoupling by UCP2 did not seem to be associated with decreased insulin secretion in βUCP2Tg islets. We further investigated the effect of genipin on mitochondrial function (Figures 5J–5L). Mitochondrial respiration after treatment with glucose or FCCP and ATP production were decreased by adenoviral expression of *Ucp2* in mouse islets (Figures 5J–5L). Proton leakage was not changed by adenoviral overexpression of *Ucp2* in mouse islets (Figure 5L), similar to βUCP2Tg islets (Figure 3C). Moreover, genipin did not affect mitochondrial respiration, proton leakage, or ATP production in UCP2-overexpressing islets (Figures 5K and 5L). From these results, UCP2 induced β-cell dysfunction without any changes of proton leakage.

### UCP2 regulated AldB expression through the oxidative-stress-mediated pathway

**Figure 6:**
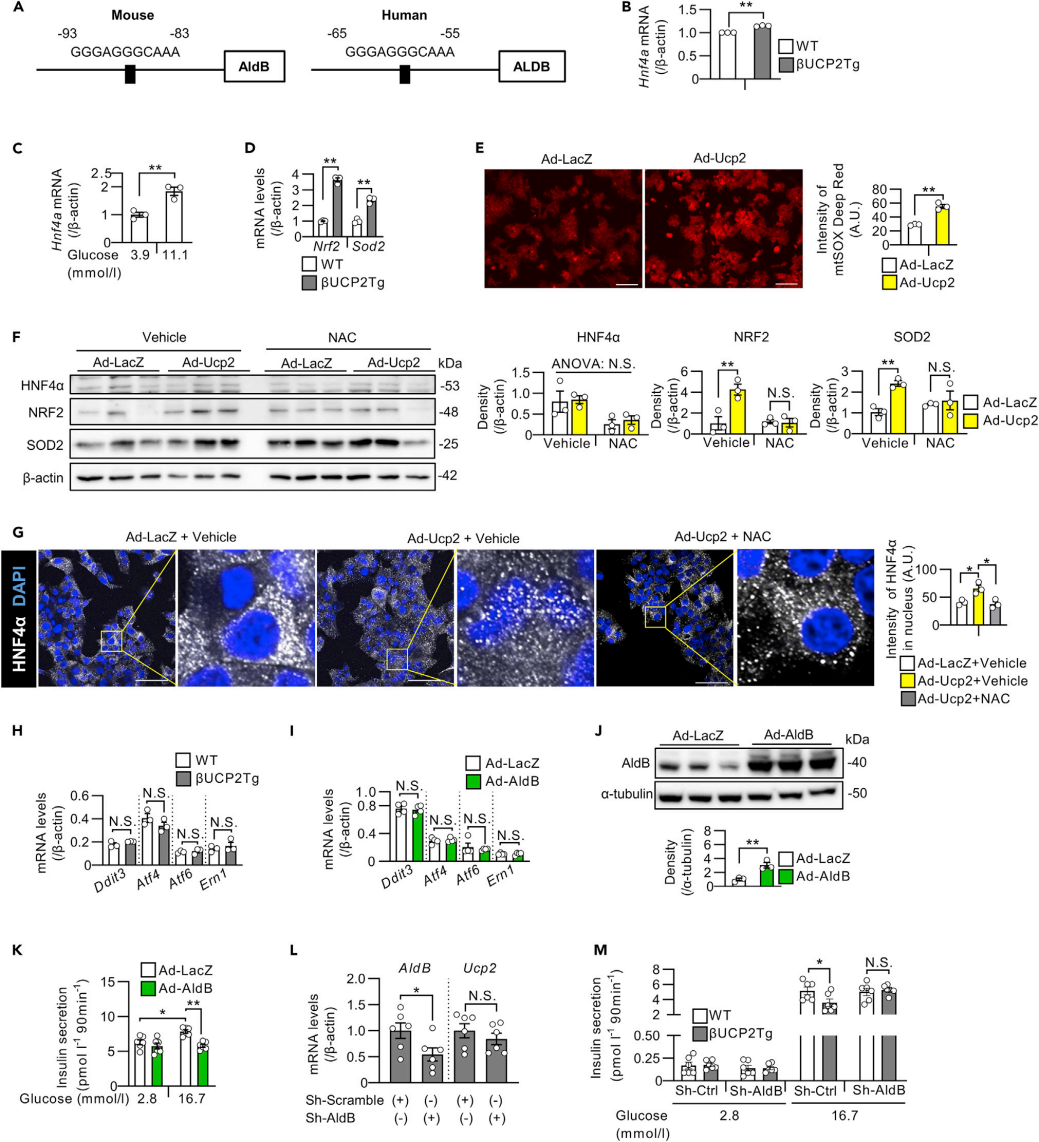
AldB was induced by UCP2 in an oxidative stress-mediated pathway (A) Schematic of the Hnf4α-binding motif in the AldB promoter region (left panel: mouse, right panel: human). (B) *Hnf4a* mRNA levels in βUCP2Tg and WT islets (n = 3 per group). Islets were incubated with 11.1 mmol/L glucose for 24 h. (C) *Hnf4a* mRNA levels in mouse islets. Islets from C57BL6/J mice were incubated with 3.9 or 11.1 mmol/L glucose for 24 h (n = 3 per group). (D) *Nrf2* and *Sod2* mRNA levels in βUCP2Tg and WT islets. Islets were incubated with 5.6 mmol/L glucose for 24 h (n = 3 per group). (E) Mitochondrial superoxide levels in MIN6-M9 cells. Cells were infected with Ad-LacZ or Ad-AldB at an MOI of 500 for 48 h. The graph shows the intensities of mtSOX Deep Red Dye in MIN6-M9 cells. The scale bar indicates 100 μm. (F) Western blot of HNF4α, NRF2, SOD2, and β-actin in mouse islets. Islets were infected with Ad-LacZ or Ad-Ucp2 at 1 × 10^4^ MOI at 11.1 mmol/L glucose in the presence or absence of NAC for 48 h (n = 3 per group). (G) Immunostaining of HNF4α and DAPI in MIN6-M9 cells. Cells were infected with Ad-LacZ or Ad-Ucp2 at an MOI of 500 for 48 h in the presence or absence of 20 mmol/L NAC. The scale bar represents 50 μm. The graph shows the intensity of HNF4α in the nucleus measured by ImageJ software. (H) *Ddit3, Atf4, Atf6*, and *Ern1* mRNA levels in βUCP2Tg and WT islets. Islets were incubated with 5.6 mmol/L glucose for 24 h (n = 3 per group). (I) *Ddit3, Atf4, Atf6*, and *Ern1* mRNA levels in mouse islets. Islets were infected with Ad-LacZ or Ad-AldB at 3 × 10^6^ MOI in the presence of 5.6 mmol/L glucose for 48 h (n = 4 per group). (J and K) Western blot of AldB and α-tubulin (J, n = 3 per group) and GSIS (K, n = 6 per group) in MIN6-M9 cells. Cells were infected with Ad-LacZ or Ad-AldB at an MOI of 500 for 48 h. For GSIS, cells were incubated in KRB buffer containing 2.8 and 16.7 mmol/L glucose for 90 min. (L) *AldB* and *Ucp2* mRNA levels in βUCP2Tg islets. Islets were infected with Sh-Scramble or Sh-AldB at 1 × 10^4^ MOI in the presence of 5.6 mmol/L glucose for 48 h (n = 6 per group). (M) GSIS in mouse islets (n = 6 per group). Islets were infected with Sh-Scramble (indicated as Sh-Ctrl) or Sh-AldB at an MOI of 1 × 10^4^ in the presence of 5.6 mmol/L glucose for 24 h. For GSIS, islets were incubated in KRB buffer containing 2.8 and 16.7 mmol/L glucose for 90 min. Data are the means ± SEM ∗p < 0.05, ∗∗p < 0.01. N.S.: not significant. The two-tailed Student’s t test was used in (B–E), (H–J), and (L). One-way ANOVA was used in (F), (G), (K), and (M).

We further investigated the regulatory mechanism of AldB expression by UCP2. Previous reports have shown that AldB is the target gene of hepatocyte nuclear factor 4α (HNF4α) in INS1 rat β-cell lines (Wang et al., 2000). We analyzed the 2-kb sequences of the murine and human AldB promoter regions and identified a putative HNF4α binding site (Figure 6A). We also found that the expression of *Hnf4a* was upregulated in βUCP2Tg islets (Figure 6B) and in WT islets exposed to high glucose (Figure 6C). The expression of *Nrf2* and *Sod2*, which are oxidative-stress-related genes, was increased in βUCP2Tg islets (Figure 6D). The signal intensity of mtSOX Deep Red, which emits fluorescence in response to mitochondrial superoxide, was increased by overexpression of UCP2 in MIN6-M9 cells (Figure 6E). We treated UCP2-overexpressing islets with NAC to evaluate the involvement of oxidative stress, and increased expression of NRF2 and SOD2 were abolished by NAC (Figure 6F). Although the protein levels of HNF4α were not significantly changed by overexpression of UCP2 in islets, the fluorescent intensity of HNF4α in the nucleus was increased by highly expressed UCP2 in MIN6-M9 cells, and this effect was reversed by NAC treatment (Figure 6G). We also investigated whether ER stress contributed to AldB induction by UCP2 because thapsigargin increased the expression of *AldB* in mouse islets (Figure 5G). As shown in Figure S1A, *Ucp2* expression was not induced by thapsigargin, and the expression of *Ddit3*, *Atf4*, *Atf6*, and *Ern1*, which are ER-stress-related genes, was not changed in βUCP2Tg islets (Figure 6H). Furthermore, adenoviral expression of *AldB* in islets did not affect the expression levels of ER-stress-related genes (Figure 6I). Based on these results, oxidative stress, but not ER stress, was involved in the increase in *AldB* expression in βUCP2Tg β-cells.

We also analyzed whether AldB participates in β-cell function. Importantly, adenovirus-vector-induced expression of *AldB* in MIN6-M9 cells decreased GSIS (Figures 6J and 6K). To investigate whether UCP2-induced AldB expression is involved in the impaired insulin secretion in βUCP2Tg mice, we conducted AldB-knockdown experiments by using an adenovirus vector that expressed sh-RNA for *AldB*. Sh-AldB reduced the *AldB* mRNA levels in βUCP2Tg islets by approximately 50% (Figure 6L). The knockdown of AldB restored GSIS in βUCP2Tg islets (Figure 6M); therefore, this result indicated that AldB expression was crucial in impaired insulin secretion in βUCP2Tg mice.

Collectively, in stressed β-cells under diabetes, mitochondrial dysfunction caused by elevated UCP2 induced oxidative stress, resulting in activation of HNF4α and thus leading to the elevation of AldB expression. AldB in turn was involved in the impaired insulin secretion caused by UCP2 upregulation.

### AldB expression impaired mitochondrial function in β-cells

**Figure 7:**
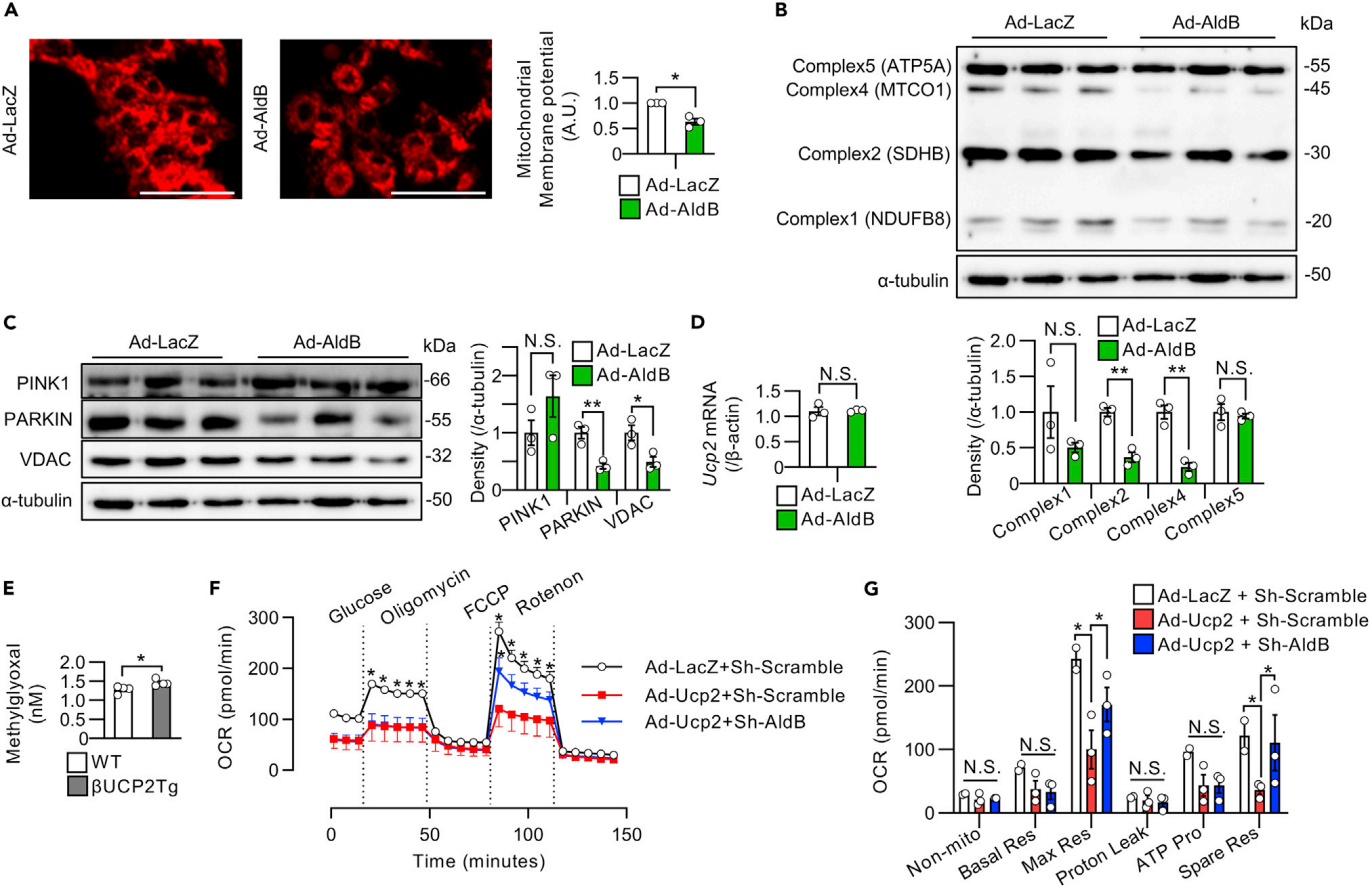
AldB impaired mitochondrial function in β-cells (A) Mitochondrial membrane potential in MIN6-M9 cells. Cells were infected with Ad-LacZ or Ad-AldB at an MOI of 500 for 48 h. The graph shows the intensities of MT-1 dye in MIN6-M9 cells. The scale bar indicates 100 μm. (B and C) Western blots of mitochondrial OXPHOS proteins and α-tubulin (B, n = 3 per group) and mitophagy-associated proteins and α-tubulin (C, n = 3 per group) in islets from C57BL/6J mice. Islets were infected with Ad-LacZ or Ad-AldB at 3 × 10^6^ MOI in the presence of 5.6 mmol/L glucose for 48 h. (D) *Ucp2* mRNA levels in islets from C57BL/6J mice. Islets were infected with Ad-LacZ or Ad-AldB at an MOI of 3 × 10^6^ in the presence of 5.6 mmol/L glucose for 48 h (n = 3 per group). (E) Methylglyoxal levels in βUCP2Tg and WT islets (n = 3 per group). Islets were incubated with 11.1 mmol/L glucose for 24 h. (F and G) Oxygen consumption rate in islets from C57BL/6J mice. Islets were infected with (1) Ad-LacZ + Sh-Scramble (n = 2), (2) Ad-Ucp2 + Sh-Scramble (n = 3), or (3) Ad-Ucp2 + Sh-AldB (n = 3) at an MOI of 1 × 10^4^ in the presence of 5.6 mmol/L glucose for 24 h. (F) The asterisk indicates a significant difference in comparison with the Ad-Ucp2 + Sh-Scramble group. (G) The graph shows nonmitochondrial oxygen consumption, basal respiration, maximal respiration, proton leakage, ATP production, and spare respiratory capacity. Data are the means ± SEM ∗p < 0.05, ∗∗p < 0.01. N.S.: not significant. The two-tailed Student’s t test was used in (A–E). One-way ANOVA was used in (F) and (G).

Next, we investigated the involvement of AldB in the mitochondrial dysfunction observed in βUCP2Tg islets. Forced expression of *AldB*, as well as *Ucp2*, in MIN6-M9 cells reduced the mitochondrial membrane potential (Figure 7A). Overexpression of *AldB* in mouse islets decreased the expression of mitochondrial OXPHOS proteins, complex 2 and complex 4, PARKIN and VDAC (Figures 7B and 7C). We also confirmed that adenovirus-vector-induced *AldB* expression in islets had no effect on *Ucp2* expression (Figure 7D). Methylglyoxal, a major precursor of advanced glycation end products, was reportedly induced by overexpression of AldB and involved in mitochondrial dysfunction in pancreatic β-cells (Bo et al., 2016; Liu et al., 2012). We found increased levels of methylglyoxal in βUCP2Tg islets (Figure 7E). Importantly, knockdown of AldB ameliorated the impairment of mitochondrial respiration induced by overexpression of UCP2 in islets (Figures 7F and 7G). These results indicated that mitochondrial dysfunction in βUCP2Tg islets was mediated, at least in part, by altered AldB expression.

### UCP2 and AldB reduce insulin secretion through dysregulation of Ca^2+^ release from the ER in β-cells

**Figure 8:**
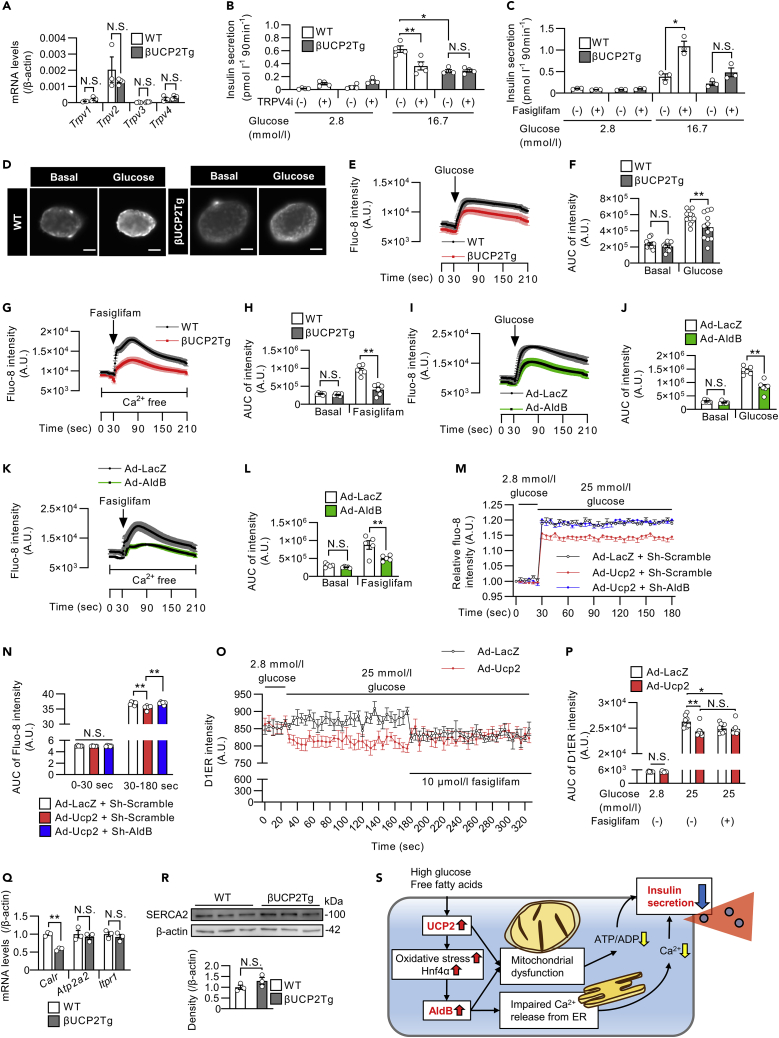
UCP2 and AldB attenuated insulin secretion through the dysregulation of Ca^2+^ release from the ER in β-cells (A) mRNA levels of *Trpv1*-*4* in βUCP2Tg and WT islets. Islets were incubated with 11.1 mmol/L glucose for 24 h (WT: n = 3, βUCP2Tg: n = 4). (B and C) GSIS in βUCP2Tg and WT islets. Islets were incubated in KRB buffer containing 2.8 and 16.7 mmol/L glucose for 90 min, with or without 100 nmol/L TRPV4 inhibitor (B, n = 4 per group), and with or without 10 μmol/L fasiglifam (C, n = 3 per group). (D–H) Intracellular Ca^2+^ imaging in βUCP2Tg and WT islets. (D) Images of the islets before and after glucose stimulation. The scale bar indicates 50 μm. (E) Fluo-8 intensity in islets was measured for 210 s. Islets were incubated with 2.8 mmol/L glucose, and then 25 mmol/L glucose was added at 30 s (WT: n = 13, βUCP2Tg: n = 12). (F) The AUC of the Fluo-8 intensity in islets before and after glucose stimulation (WT: n = 13, βUCP2Tg: n = 12). (G) Fluo-8 intensity in islets was measured for 210 s. Islets were incubated in Ca^2+−^free medium, and then 100 μmol/L fasiglifam was added at 30 s (n = 6 per group). (H) The AUC of Fluo-8 intensity in islets before and after fasiglifam stimulation (n = 6 per group). (I–L) Intracellular Ca^2+^ imaging in islets infected with Ad-LacZ or Ad-AldB (3 × 10^6^ MOI) for 24 h using the same procedure as that shown in D–H. (I and K) Fluo-8 intensity (I: n = 6 per group, K: n = 5 per group). (J and L) AUC of Fluo-8 intensity (J: n = 6 per group, L: n = 5 per group). (M and N) Intracellular Ca^2+^ concentrations in INS1 β-cell lines infected with (1) Ad-LacZ + Sh-Scramble, (2) Ad-Ucp2 + Sh-Scramble, or (3) Ad-Ucp2 + Sh-AldB at an MOI of 10 in the presence of 11.1 mmol/L glucose for 48 h. The fluo-8 intensity in the cells was measured every 5 s for 180 s by using a fluorescence microplate reader. Cells were stimulated with 25 mmol/L glucose medium at 30 s (n = 6 per group). The presented data are the ratio to the value from INS1 cells infected with Ad-LacZ control. (N) The AUC of the Fluo-8 intensity in INS1 cells before and after glucose stimulation (n = 6 per group). (O and P) Ca^2+^ concentrations in the ER in INS1 β-cell lines infected with Ad-LacZ or Ad-Ucp2 at an MOI of 10 in the presence of 11.1 mmol/L glucose for 48 h (n = 8 per group). The D1ER plasmid, an ER Ca^2+^ reporter, was transfected into cells 24 h before Ca^2+^ measurement. Two hours prior to measurement, the medium was changed to HBSS containing 2.8 mmol/L glucose. The intensity of the D1ER was measured every 5 s by a fluorescence microplate reader. Then, 25 mmol/L glucose was added at 30 s, and 10 μmol/L fasiglifam was added at 180 s. D1ER was excited at 440 nm with emission at 485 nm. (P) AUC of D1ER intensity in INS1 cells at 0–30 s, 30–180 s, and 180–330 s (n = 8 per group). (Q) mRNA levels of *Calr*, *Atp2a2*, and *Itpr1* in βUCP2Tg and WT islets. Islets were incubated with 11.1 mmol/L glucose for 8 h (n = 3 per group). (R) Western blot of SERCA2 and β-actin in βUCP2Tg and WT islets. Islets were incubated with 5.6 mmol/L glucose for 24 h (n = 3 per group). (S) Schematic of the mechanism by which the upregulation of UCP2 and AldB impairs insulin secretion from β-cells in type 2 diabetes. Data are the means ± SEM ∗p < 0.05, ∗∗p < 0.01. N.S.: not significant. The two-tailed Student’s t test was used in (A), (Q), and (R). One-way ANOVA was used in (B), (C), (F), (H), (J), (L), (N), and (P).

Li et al. reported that the aldolase enzyme inhibits a TRPV4 channel to restrict Ca^2+^ release from the ER under glucose starvation conditions in mouse embryonic fibroblasts (Li et al., 2019). Therefore, we investigated the roles of UCP2 and AldB in Ca^2+^ release from the ER in β-cells. We confirmed the expression of *Trpv2* and *Trpv4* in mouse islets (Figure 8A). HC 067047, a selective TRPV4 inhibitor, decreased GSIS in WT islets but not in βUCP2Tg islets (Figure 8B). Fasiglifam (TAK-875), a GPR40 agonist, has been shown to enhance insulin secretion from β-cells through inositol trisphosphate (IP3)-mediated Ca^2+^ release from the ER (Nolan et al., 2006). In our study, fasiglifam-induced enhancement of insulin secretion was attenuated in βUCP2Tg islets (Figure 8C). These results suggested that UCP2 participated in the regulation of Ca^2+^ release from the ER in pancreatic β-cells.

We assessed intracellular Ca^2+^ influx in βUCP2Tg islets. The increase in intracellular Ca^2+^ by glucose was attenuated in βUCP2Tg islets compared with islets from WT mice (Figures 8D–8F). The fasiglifam-induced increase in intracellular Ca^2+^, after depletion of extracellular Ca^2+^ levels, was completely blunted in βUCP2Tg islets, indicating that Ca^2+^ release from the ER by fasiglifam was attenuated in βUCP2Tg islets (Figures 8G and 8H). Furthermore, adenovirus-vector-induced expression of *AldB* in mouse islets also resulted in impaired Ca^2+^ influx in response to glucose or fasiglifam (Figures 8I–8L). These results indicated that overexpression of UCP2 and the consequent induction of AldB expression in mouse islets resulted in impaired Ca^2+^ release from the ER and reduced insulin secretion from pancreatic islets. The knockdown of AldB ameliorated the dysregulation of glucose-stimulated intracellular Ca^2+^ influx caused by UCP2 overexpression in INS1 β-cell lines (Figures 8M and 8N). We further evaluated the Ca^2+^ concentration in the ER using the FRET-based probe D1ER cameleon (Palmer et al., 2004). The ER Ca^2+^ concentration in control INS1 cells slightly increased after high glucose exposure, whereas UCP2-overexpressing INS1 cells exhibited decreased ER Ca^2+^ (Figures 8O and 8P). We also found that the reduction in Ca^2+^ concentration in the ER after fasiglifam stimulation was not recognized by overexpression of UCP2 in INS1 β-cell lines (Figures 8O and 8P). Although the expression levels of the Ca^2+^-binding ER protein *Calr* were decreased in βUCP2Tg islets, there were no changes in the mRNA expression of the Ca^2+^-ATPase pump *Atp2a2*, which encodes the SERCA2 protein involved in ER Ca^2+^ influx or the ER Ca^2+^ efflux channel *Itpr1* (Figure 8Q). The protein levels of SERCA2 also showed no changes in βUCP2Tg islets (Figure 8R). Altogether, UCP2-mediated AldB expression might induce the dysregulation of Ca^2+^ release from the ER in pancreatic β-cells.

## Discussion

In this study, we report that an increase in UCP2 expression in pancreatic β-cells induced AldB expression, which in turn impaired insulin secretion by promoting mitochondrial dysfunction and impaired Ca^2+^ release from the ER (Figure 8S).

The induction of UCP2 by high glucose or oxidative stress is consistent with several independent reports that used INS-1 β-cells, rat islets, and human islets (Brown et al., 2002; Krauss et al., 2003; Li et al., 2009; Medvedev et al., 2002; Pi et al., 2009). In the present study, we report that Akt and ERK, which are activated by glucose, are also involved in UCP2 expression in β-cells. Meanwhile, UCP2 has been shown to be upregulated by glucotoxicity in GLUTag cells, an intestinal L-cell model, and to reduce Glp-1 secretion from GLUTag cells, suggesting that UCP2 contributes to the pathogenesis of T2DM not only in β-cells (Urbano et al., 2016). Further studies are required to clarify the factors that determine UCP2 expression during the development of diabetes.

The impact of UCP2 overexpression in islets on insulin secretion *in vitro* remains controversial (Chan et al., 1999; Produit-Zengaffinen et al., 2007). In the current study, βUCP2Tg mice exhibited reduced insulin secretion early (5 min) after glucose loading but not at later time points (e.g., 15 min). Thus, UCP2 might be important in the early secretory response to glucose. The results of Ca^2+^ imaging also showed that early elevation of intracellular Ca^2+^ influx after glucose stimulation (e.g., 20 s) was attenuated in βUCP2Tg islets. We also explored the effect of the GLP-1 agonist liraglutide on GSIS in βUCP2Tg islets. GLP-1R signaling increases intracellular cAMP levels, which activate protein kinase A (PKA) and Epac2, resulting in enhanced insulin secretion in β-cells (Holst, 2007). Our data showed that liraglutide increased insulin secretion in βUCP2Tg islets as well as WT islets. Therefore, it was considered that the enhancement of insulin secretion through the GLP-1R pathway was not diminished in βUCP2Tg islets.

Dilatation of the mitochondria and decreased expression of OXPHOS proteins in the mitochondria were observed in βUCP2Tg β-cells in association with reduced ATP production. Because mitochondrial respiration was impaired without any change in proton leakage in βUCP2Tg islets, the deterioration of the electron transport chain may affect the result rather than the uncoupling. It has been shown that mitochondrial complex protein was reduced in β-cells from diabetic mice (Brown et al., 2021; Haythorne et al., 2019). Haythorne et al. also demonstrated that the protein levels of NDUFB8 were decreased in islets from diabetic βV59M mice, but its mRNA levels were not changed, thus the downregulation of NDUFB8 protein may occur at the posttranslational level under diabetic conditions. These reports were consistent with our data, where NDUFB8 protein levels were decreased with no changes in mRNA levels in βUCP2Tg islets.

Dilatation of the mitochondria in β-cells has also been observed in diabetic db/db mice (Lo et al., 2010). Although the protein level of PARKIN, an E3 ubiquitin ligase that plays a crucial role in mitophagy, was decreased in βUCP2Tg islets, mitophagy flux was increased. The deletion of PARKIN in pancreatic β-cells did not affect insulin secretion (Corsa et al., 2019). From these findings, it was considered that altered mitophagy-related protein levels in βUCP2Tg islets did not contribute to impaired insulin secretion and that decreased PARKIN expression was caused by the adaptive response of mitophagy and the consumption of PARKIN proteins.

We focused on the role of AldB in β-cells because its expression level was markedly upregulated in βUCP2Tg islets. Notably, the expression of both UCP2 and AldB has been reported to be increased in the islets of T2DM donors (Anello et al., 2005; Gerst et al., 2018). The expression of AldB in human islets has been reported to be negatively associated with insulin secretion in subjects with T2DM (Gerst et al., 2018). Indeed, the expression of AldB was increased in islets from diabetic db/db and IRS2KO mice, and overexpression of AldB attenuated insulin secretion in this study. We also showed an increase in the expression of AldB in human islets in response to high glucose.

We showed that oxidative stress and HNF4α might be involved in AldB transcription. HNF4α is a nuclear receptor (Nammo et al., 2008), and its binding to DNA is inhibited by AICAR, an activator of AMPK (Hong et al., 2003). The expression of downstream genes of HNF4α was suppressed by reduced nuclear localization of HNF4α in human hepatoma cells (Chandra et al., 2011). Therefore, in β-cells from βUCP2Tg mice, inactivation of AMPK by oxidative stress possibly increased the DNA binding of HNF4α and the nuclear translocation of HNF4α, which resulted in upregulation of AldB transcription. Dephosphorylation of AMPK is reportedly induced by oxidative stress in C2C12 mouse myoblast cell lines (Jiang et al., 2021). The intensity of mtSOX Deep Red, which is an indicator of mitochondrial oxidative stress, and NRF2 and SOD2 expression were increased by adenoviral expression of *Ucp2* in MIN6-M9 cells or islets. Thus, the excessive UCP2 induced by glucose-induced oxidative stress might further potentiate oxidative stress that leads to *AldB* expression. A previous report showed the protective effect of UCP2 against oxidative stress in β-cells (Li et al., 2017). However, they also showed that superoxide levels at both 11 mmol/L and 30 mmol/L glucose tended to be increased in UCP2-overexpressing islets. Their data indicate that oxidative stress is induced by UCP2 and are consistent with our data demonstrating the upregulation of NRF2 and SOD2 in βUCP2Tg islets.

Although it was reported that activation of NRF2 promoted mitochondrial biogenesis and insulin secretion in β-cells (Kumar et al., 2018; Yagishita et al., 2014), our UCP2 transgenic mice did not show any increase in insulin secretion. It was unclear whether the increased expression of NRF2 was involved in the phenotype of UCP2 transgenic mice, but this NRF2 upregulation was considered insufficient to restore the decreased insulin secretion in βUCP2Tg mice. Overexpression of AldB in islets reduced the mitochondrial protein levels of OXPHOS, VDAC, and PARKIN, suggesting the involvement of AldB in mitochondrial dysfunction in βUCP2Tg islets. In fact, the increase in AldB expression in MIN6-M9 cells resulted in decreased mitochondrial membrane potential. In previous reports, knockdown of AldB in endothelial cells ameliorated the high glucose-induced accumulation of methylglyoxal (Liu et al., 2012), which is known to induce mitochondrial dysfunction (Bo et al., 2016). AldB is the most upregulated protein in the islets of diabetic βV59M mice, and pathway analysis of the transcriptome data revealed a downregulation of OXPHOS in the mitochondria (Haythorne et al., 2019). Fructose 1,6-bisphosphatase 1 (FBP1), a protein that interacts with AldB, is upregulated in the β-cells of T2DM mice, and inhibition of FBP1 improves insulin secretion (Zhang et al., 2010). Taken together, AldB expression may mediate mitochondrial dysfunction, and decreased insulin secretion was observed in βUCP2Tg mice.

Our results indicated that UCP2 and AldB impaired Ca^2+^ release from the ER in β-cells. Moreover, in mouse embryonic fibroblasts, aldolase binds to the TRPV4 channel under glucose starvation conditions, following activation of AMPK phosphorylation (Li et al., 2019). Because the effects of a selective TRPV4 inhibitor or fasiglifam on insulin secretion and Ca^2+^ influx are blunted in islets overexpressing UCP2 or AldB, it is possible that UCP2 or AldB inhibits TRPV channel activity and Ca^2+^ release from the ER in pancreatic β-cells. The interaction of aldolase and TRPV4 channels has been shown (Li et al., 2019). Hence, AldB might modulate the activity of TRPV4 channels. Because the intracellular Ca^2+^ influx through TRPV4 channels reportedly induced IP3-mediated Ca^2+^ release from the ER in endothelial cells (Heathcote et al., 2019), the interaction of TRPV4 and AldB might affect insulin secretion in βUCP2Tg β-cells through IP3-mediated Ca^2+^ release. Ca^2+^ release from the ER is also mediated by ryanodine receptors (RyR), and pharmacological inhibition of RyR reduces GSIS (Llanos et al., 2015). Ca^2+^ release from the ER induced by GLP-1R signaling is blocked by the inhibition of RyR but not by the IP3 receptor antagonist in mouse islets and INS1 β-cell lines (Kang et al., 2001; Kim et al., 2008). Our data showed that enhancement of insulin secretion by liraglutide was preserved in βUCP2Tg islets. Therefore, it was considered that RyR-mediated Ca^2+^ release from the ER was not involved in the decreased insulin secretion in βUCP2Tg β-cells.

GPR40 agonists reportedly enhanced GSIS through IP3-mediated Ca^2+^ release from the ER- and diacylglycerol (DAG)-mediated activation of protein kinase C (PKC) (Yamada et al., 2016). Because the Ca^2+^ concentration in the ER after stimulation with the GPR40 agonist was not decreased in UCP2-overexpressing INS1 cells, IP3-mediated Ca^2+^ efflux from the ER was impaired by excessive UCP2. We also found that the Ca^2+^ concentration in the ER was decreased after glucose stimulation by overexpression of UCP2 in INS1 cells. Because methylglyoxal has been shown to impair the activity of SERCA, a calcium pump in the ER, without any changes of protein levels of SERCA (Zizkova et al., 2018), increased methylglyoxal in βUCP2Tg islets might decrease ER Ca^2+^ uptake through SERCA. In MIN6 mouse β-cell lines, Ca^2+^ uptake through SERCA after high glucose stimulation was inhibited by oligomycin, an inhibitor of ATP synthase (Moore et al., 2011), thus the reduction in ATP production in βUCP2Tg islets might affect SERCA activity. Because UCP2 was also shown to act as a Ca^2+^ uniporter in mitochondria (Koshenov et al., 2020; Trenker et al., 2007), the decrease in the ER Ca^2+^ concentration after glucose stimulation in UCP2-overexpressing INS1 cells might be caused by increased Ca^2+^ uptake through UCP2.

We also showed decreased expression of *Calr* in βUCP2Tg islets. Knockdown of calreticulin in MEFs reduced Ca^2+^ storage in the ER and impaired IP3-dependent Ca^2+^ release from the ER (Mesaeli et al., 1999). However, the basal Ca^2+^ concentration in the ER was not changed by UCP2 overexpression in INS1 cells. Because increased calreticulin was reported to inhibit Ca^2+^ release from the ER because of its high affinity for Ca^2+^ in *C. elegans* (Burkewitz et al., 2020), the reduced *Calr* expression in βUCP2Tg islets might be the adaptive response against the impairment of Ca^2+^ release from the ER. Taken together, our data suggest that the impairment of Ca^2+^ release from the ER reduces GSIS in βUCP2Tg β-cells.

Given the role of UCP2 in Ca^2+^ uptake by mitochondria in HeLa cells (Madreiter-Sokolowski et al., 2016), it is possible that impaired Ca^2+^ influx in βUCP2Tg β-cells is, at least in part, due to an upregulation of mitochondrial Ca^2+^ uptake. Whether UCP2 has a role as a classic uncoupling protein, such as UCP1, is controversial (Nicholls, 2016). A previous report showed that overexpression of UCP2 does not reduce the mitochondrial membrane potential in rat INS-1 β-cells, suggesting that UCP2 does not act as an uncoupler (Galetti et al., 2009). Indeed, proton leakage in βUCP2Tg islets was not changed compared with that in WT islets. UCP2 has a half-life of 30 min, whereas the half-life of UCP1 is 30 h (Rousset et al., 2007). This difference in half-life among UCP proteins also indicates distinct physiological functions of UCP2. A previous report characterized UCP2 as a metabolite transporter capable of reducing glucose oxidation in mitochondria (Vozza et al., 2014). Our data also demonstrated a reduction in ATP production and reduced mitochondrial respiration in βUCP2Tg islets. Because decreased GSIS, reduced mitochondrial respiration, and impairment of Ca^2+^ flux by excessive UCP2 in β-cells were ameliorated by AldB knockdown, UCP2-induced AldB contributed to the β-cell dysfunction of βUCP2Tg mice. In particular, AldB-mediated intracellular Ca^2+^ regulation by UCP2 in our study may provide clues to the novel pathologic mechanisms underlying β-cell failure.

In summary, we provide evidence that the upregulation of UCP2 in stressed β-cells under diabetes decreased insulin secretion due to mitochondrial dysfunction and impairment of Ca^2+^ release from the ER by inducing AldB expression. Therefore, inhibition of UCP2 or AldB might be a promising therapeutic strategy to improve insulin secretion in patients with diabetes.

### Limitations of the study

Our study demonstrates the molecular role of UCP2 and AldB as regulators of insulin secretion in β-cells. We have demonstrated that UCP2 induces AldB expression and suppresses insulin release via dysfunction in mitochondria or Ca^2+^ release from the ER. Although we showed the requirement of AldB for impaired insulin secretion in UCP2-overexpressing β-cells in *in vitro* or *ex vivo* experiments, it was not evaluated *in vivo*. An inducible β-cell-specific AldB conditional knockout model will be a convincing tool to clarify this issue. This study highlights the expression and roles of UCP2 and AldB in human islets. To confirm the pathological or therapeutic significance of these two genes, a study using islets from donors with type 2 diabetes needs to be performed. Due to the unavailability and the extensive phenotypic diversity in type 2 diabetes islets, further studies will be needed.

## STAR★Methods

### Key resources table

**Table:** 

REAGENT or RESOURCE	SOURCE	IDENTIFIER
**Antibodies**		

Alpha Tubulin antibody[DM1A]	Abcam	Cat# ab7291; RRID: AB_2241126
Anti-beta Actin antibody	Abcam	Cat# ab8227; RRID: AB_2305186
UCP2 (D1O5V) Rabbit mAb	Cell Signaling Technology	Cat# 89326; RRID: AB_2721818
UCP2 (C-20) antibody	Santa Cruz Biotechnology	Cat# sc-6525; RRID: AB_2213585
UCP2 Polyclonal antibody	Proteintech	Cat# 11081-1-AP; RRID: AB_2213793
ALDOB antibody	Proteintech	Cat# 18065-1-AP; RRID: AB_2273968
PINK1 antibody	Abcam	Cat# ab23707; RRID: AB_447627
Parkin (PRK8) antibody	Santa Cruz Biotechnology	Cat# sc-32282; RRID: AB_628104
VDAC antibody	Cell Signaling Technology	Cat# 4866; RRID: AB_2272627
Total OXPHOS Rodent WB Antibody Cocktail	Abcam	Cat# ab110413; RRID: AB_2629281
SOD2 anti body	Proteintech	Cat# 24127-1-AP; RRID: AB_2879437
NRF2 anti body	Proteintech	Cat# 16396-1-AP; RRID: AB_2782956
HNF4-alpha antibody	Abcam	Cat# ab201460; RRID: N/A
Insulin (H-86) antibody	Santa Cruz Biotechnology	Cat# sc-9168; RRID: AB_2126540
anti-bromodeoxyuridine, anti-BrdU antibody	Dako	Cat# M0744; RRID: AB_10013660
Guinea Pig Anti-insulin Polyclonal Antibody	Abcam	Cat# ab7842; RRID: AB_306130
Donkey Anti-Goat IgG H&L (Alexa Fluor® 488)	Abcam	Cat# ab150129; RRID: AB_2687506
Donkey anti-Goat IgG (H + L) Cross-Adsorbed Secondary Antibody, Alexa Fluor 647	Invitrogen	Cat# A-21447; RRID: AB_141844
Alexa Fluor® 594 AffiniPure Donkey Anti-Guinea Pig IgG (H + L)	Jackson ImmunoResearch	Cat# 706-585-148; RRID: AB_2340474
Donkey anti-Rabbit IgG (H + L) Highly Cross-Adsorbed Secondary Antibody, Alexa Fluor 488	Invitrogen	Cat# A-21206; RRID: AB_141708
Donkey anti-Rabbit IgG (H + L) Highly Cross-Adsorbed Secondary Antibody, Alexa Fluor 555	Invitrogen	Cat# A-31572; RRID: AB_162543

**Bacterial and virus strains**		

Ad-GFP-U6-m-ALDOB-shRNA	VECTOR BIOLABS	Cat# shADV-252486

**Biological samples**		

Human islets, see Table S1	Alberta Islet Distribution Program and Islet Core	https://www.epicore.ualberta.ca/isletcore/

**Chemicals, peptides, and recombinant proteins**		

RPMI 1640(No Glucose) with L-Gln, liquid	Nacalai tesque, Kyoto, Japan	Cat# 09892-15
DMEM(No Glucose) with L-Gln, without Sodium Pyruvate, liquid	Nacalai tesque, Kyoto, Japan	Cat# 09891-25
Agilent Seahorse XF RPMI Medium (without Phenol Red)	Agilent Technologies	Cat# 103336-100
HBSS(+) with Ca, Mg, without Phenol Red, liquid	Nacalai tesque, Kyoto, Japan	Cat# 09735-75
HBSS(-) without Ca, Mg and Phenol Red, liquid	Nacalai tesque, Kyoto, Japan	Cat# 17461-05
D-PBS(-) without Ca and Mg, liquid	Nacalai tesque, Kyoto, Japan	Cat# 14249-95
Final Wash/Culture Medium	Mediatech	Ca# 99-785-CV
45(w/v)%-D-(+)-Glucose Solution	Nacalai tesque, Kyoto, Japan	Cat# G8769
100mM-Sodium Pyruvate Solution(100x)	Nacalai tesque, Kyoto, Japan	Cat# 06977-34
Penicillin-Streptomycin Mixed Solution	Nacalai tesque, Kyoto, Japan	Cat# 26253-84
200mmol/l L-Alanyl-L-glutamine Solution(100x)	Nacalai tesque, Kyoto, Japan	Cat# 04260-64
1mol/L-HEPES Buffer Solution	Nacalai tesque, Kyoto, Japan	Cat# 17557-94
2-Mercaptoethanol	Nacalai tesque, Kyoto, Japan	Cat# 21417-52
5-Bromo-2′-deoxyuridine	Nacalai tesque, Kyoto, Japan	Cat# 05650-66
Poly-L-lysine solution	Sigma-Aldrich	Cat# P4707
Protease Inhibitor Cocktail(EDTA free) (100x)	Nacalai tesque, Kyoto, Japan	Cat# 03969-21
Phosphatase Inhibitor Cocktail(EDTA free) (100x)	Nacalai tesque, Kyoto, Japan	Cat# 07575-51
SYBR® Green Supermix	Bio-Rad	Cat# 1725274
METAFECTENE® PRO	Biontex	Cat# T040
Glucokinase Activator, CpdA	Calbiochem	Cat# 346021
Insulin solution human	Sigma-Aldrich	Cat# I9278
Humulin R	Eli Lilly	HI-210
N-acetyl-L-cysteine cell culture tested	Sigma-Aldrich	Cat# A9165
Sodium palmitate	Sigma-Aldrich	Cat# P9767
Thapsigargin	Sigma-Aldrich	Cat# T9033
Rapamycin	Calbiochem	Cat# 553210
Diazoxide	Wako Pure Chemical Industries	Cat# 364-98-7
Nifedipin	Sigma-Aldrich	Cat# N7634
FK-506	Sigma-Aldrich	Cat# F4679
Akt Inhibitor X	Calbiochem	Cat# 124020
U0126	Cell Signaling Technology	Cat# 9903S
OSI-906	Selleck Chemicals	Cat# S1091
Liraglutide	Novo Nordisk	N/A
Genipin	Wako Pure Chemical Industries	Cat# 078-03021
HC-067047	Santa Cruz Biotechnology	Cat# sc-361204
TAK-875 (Fasiglifam)	AdooQ Bio Science	Cat# A11018
DAPI solution	Wako Pure Chemical Industries	Cat# 340-07971
Opti-MEM™	Thermo Fisher Scientific	Cat# 31985062
Fluoro-KEEPER Antifade Reagent, Non-Hardening Type	Nacalai tesque, Kyoto, Japan	Cat# 12593-64
ProLong™ Gold Antifade Mountant	Invitrogen	Cat# P10144
No.1SHT(0.17 ± 0.005mm) cover glass	MATSUNAMI	N/A
No.1(0.13–0.17mm) cover glass	MATSUNAMI	Cat# C024241
Fluo-8®, AM	AAT bioquest	Cat# 21081
EGTA	Wako Pure Chemical Industries	Cat# 348-01311

**Critical commercial assays**		

Ultra Sensitive Mouse Insulin ELISA Kit	Morinaga Institute of Biological Science, Yokohama, Japan	Cat# MS303
Ultra Sensitive Human Insulin ELISA Kit	Mercodia	Cat# 10-1132-01
Methylglyoxal colorimetric assay kit	Bio Vision	Cat# K500-100
Glutest Neo Super	Sanwa Chemical Co. Kanagawa, Japan	N/A
Protein Assay BCA Kit	Nacalai tesque, Kyoto, Japan	Cat# 06385-00
RNeasy Mini Kit	QIAGEN	Cat# 74106
High-Capacity cDNA Reverse Transcription Kit	Thermo Fisher Scientific	Cat# 4368813
ADP/ATP Ratio Assay Kit	Abcam	Cat# ab65313
VECTASTAIN® Elite® ABC HRP Kit	Vector Laboratories	Cat# PK-6101
DAB Peroxidase (HRP) Substrate Kit	Vector Laboratories	Cat# SK-4100
pAd/CMV/V5-DEST™ Gateway™ Vector Kit	Invitrogen	Cat# V49320
pENTR™/D-TOPO™ Cloning Kit	Invitrogen	Cat# K240020
Seahorse XFe96 Flux Pak	Agilent Technologies	Cat# 102416-100
Seahorse XF Mito Stress Kit	Agilent Technologies	Cat# 103015-100
Mitophagy Detection Kit	Dojindo Molecular Technologies	Cat# MD01
MT-1 MitoMP Detection Kit	Dojindo Molecular Technologies	Cat# MT13
mtSOX Deep Red –Mitochondrial Superoxide Detection kit	Dojindo Molecular Technologies	Cat# MT14

**Deposited data**		

Gene expression profiling in islets from βUCP2Tg mice by array	This paper	GSE147269, https://www.ncbi.nlm.nih.gov/geo/query/acc.cgi?acc=GSE147269

**Experimental models: Cell lines**		

Control β-cells	Laboratory of R.N. Kulkarni (Assmann et al., 2009; Kulkarni et al., 1999)	N/A
IRS2KO β-cells	Laboratory of R.N. Kulkarni (Assmann et al., 2009; Kulkarni et al., 1999)	N/A
MIN6-m9 β-cells	Laboratory of Susumu Seino (Minami et al., 2000)	N/A
INS1 832/13 β-cells	Laboratory of Christopher Newgard (Hohmeier et al., 2000)	N/A

**Experimental models: Organisms/strains**		

βUCP2Tg mice	This paper	Referred to as RIP-UCP-2: Accession No. CDB0543T: http://www2.clst.riken.jp/arg/micelist.html
Mouse: BKS.Cg-Dock7m+/+Leprdb/J	Charles River Japan	https://www.crj.co.jp/product/rm/detail/db
Mouse: IRS-2-/-mice	Kubota et al. (2000)	N/A

**Oligonucleotides**		

Primers for qPCR, see Table S2	This paper	N/A

**Recombinant DNA**		

Plasmid: Aldob Mouse tagged Clone	ORIGENE	Cat# MR205585
Plasmid: UCP2 Mouse Untagged Clone	ORIGENE	Cat# MC205697
Plasmid: pcDNA-D1ER	Addgene	Cat# 36325; RRID: Addgene_36325

**Software and algorithms**		

ImageJ software	NIH	https://imagej.nih.gov/ij/
Prism 8 software	Graph Pad Software	https://www.graphpad.com/scientific-software/prism/
Image LabTM software	Bio-Rad	https://www.bio-rad.com/
Wave 2.6.0 Software	Agilent technologies	https://www.agilent.com/en/products/cell-analysis/xf-cell-mito-stress-testreport-generator
SPSS Statics 26	IBM	https://www.ibm.com
BIOREVO software	KEYENCE	https://www.keyence.co.jp/

**Other**		

Applied Biosystems 7900HT Fast Real-Time PCR System	Applied Biosystems	N/A
FluoView FV1000-D confocal laser scanning microscope	Olympus	http://olympusconfocal.com/products/fv1000/
TCS SP8 STED	Leica	https://www.leica-microsystems.com/products/confocal-microscopes/p/leica-tcs-sp8-sted-one/
Chemi Doc Touch	Bio-Rad	Cat# 1708370
All-In-One Fluorescence Microscope	KEYENCE	Model BZ-X800, BZ-9000
Transblot® Turbo™	Bio-Rad	Cat# 170-4150
Wallac 1420 ARVO mX	Perkin Elmer	N/A
Seahorse XFe96 Analyzer	Agilent Technologies	https://www.agilent.com/en/products/cell-analysis/seahorse-analyzers/seahorse-xfe96-analyzer
EnSpire Multimode Plate Reader	Parkin Elmer	N/A
Automated quantitative western blotting Abby system	ProteinSimple	https://www.proteinsimple.com/abby.html

### Resource availability

#### Lead contact

Further information and requests for resources and reagents should be directed to and will be fulfilled by the Lead Contact, Jun Shirakawa MD PhD. (jshira@gunma-u.ac.jp).

#### Materials availability

βUCP2Tg mice lines in this study have been deposited at CDB database (referred to as RIP-UCP-2: Accession No. CDB0543T: http://www2.clst.riken.jp/arg/micelist.html).

### Experimental model and subject details

#### Mice

βUCP2Tg mice (referred to as RIP-UCP-2: Accession No. CDB0543T: http://www2.clst.riken.jp/arg/micelist.html) were generated as follows. A mouse UCP2 cDNA clone was obtained from Origene (#MC205697). The transgene consisted of 801 bp of the rat *Ins2* promoter linked to an intron sequence of rabbit β-globin, the *Ucp2* cDNA and a polyadenylation sequence. The purified 2.9-kb fragment digested with Not1 was microinjected into fertilized eggs. The primers used for genotyping PCR were as follows: forward primer: 5′-CATCCTGCCTTTCTCTTTATGG-3′, reverse primer: 5′-AGGAACTTCACAGTGGCTGTTG-3′. The mice were backcrossed with C57BL6/J mice more than 10 times. 8- to 20-week-old male βUCP2Tg mice and their littermate wild-type (WT) mice were used in all experiments. Male BKS.Cg-Dock7^m+/+^Lepr^db^/J (db/db) and their controls (db/+) were obtained from Charles River Japan (Yokohama, Japan). Male insulin receptor substrate (Irs)-2-deficient (IRS2KO) mice (Kubota et al., 2000) and db/db mice were used for the islet study and those mice were 9-11 weeks old. All mice were housed under a 12-hour light-dark cycle. This study was conducted with the approval of the Animal Care Committee of Yokohama City University (Permit No. F-A-16–055) and Gunma University (Permit No. 21–031). All animal procedures were performed in accordance with the institutional animal care guidelines and the guidelines of the Animal Care Committee of Yokohama City University and Gunma University.

#### Cell lines and adenovirus

β-cell lines from male control or IRS2KO mice, INS1 832/13 cells, and MIN6-M9 cells have been described previously (Assmann et al., 2009; Hohmeier et al., 2000; Kulkarni et al., 1999; Minami et al., 2000). β-cell lines from control and IRS2KO mice were used for qPCR experiments and maintained in high-glucose (25 mmol/L) DMEM supplemented with 10% FBS and 1% penicillin/streptomycin. INS1 832/13 cells were used for western blot and intracellular Ca^2+^ assay, and maintained in RPMI1640 containing 11.1 mmol/L glucose, 10% FBS and 1% penicillin/streptomycin. MIN6-M9 cells were maintained in high-glucose (25 mmol/L) DMEM supplemented with 10% FBS and 1% penicillin/streptomycin and used for the experiments of qPCR, western blot, insulin secretion assay, mitochondrial membrane potential, mitochondrial oxidative stress and immunostaining. In all experiments, cells were incubated at 37°C. Adenoviruses containing *Ucp2*, *AldB*, or *LacZ* were generated using the Virapower adenoviral expression system (Invitrogen, Switzerland) as described elsewhere (Inoue et al., 2018). Sh-RNA for mouse AldB and scramble control were purchased from VECTOR BIOLABS (USA). The sequence of shRNA was as follows. Sh-AldB: 5′-CCGGGTGAGGAGGATGCTACACTTA-CTCGAG-TAAGTGTAGCATCCTCCTCAC-TTTTTG-3′. Sh-Scramble: 5′-GATCC-AGTACTGCTTACGATACGG-TTCAAGAGA-CCGTATCGTAAGCAGTAC-TTTTTTT-3′.

#### Human islet studies

Human islets were obtained from the Alberta Islet Distribution Program and Islet Core. The details of the human islets are described in Table S1. Upon receipt, the islets were cultured overnight in Miami Medium #1A (Mediatech, USA) and then cultured in Final Wash/Culture Medium (Mediatech). Human islets were cultured at 37°C in a CO2 incubator. For the immunostaining experiments, human islets were cultured in the presence of 5.6 mmol/L or 16.7 mmol/L glucose for 48 h and then embedded in agarose. For the GSIS and qPCR study, human islets were infected with Ad-LacZ or Ad-Ucp2 at 3 × 10^6^ MOI for 48 h and then treated with glucose in Krebs-Ringer bicarbonate (KRB) buffer medium. The insulin concentration in KRB buffer and insulin content of islets were measured by using a human insulin ELISA kit (Mercodia, Sweden). All the studies and protocols for human islets were approved by the Yokohama City University Ethics Board (approval B171100025) and Gunma University Ethics Board (approval HS2020-174).

### Method details

#### Immunoblotting and Abby western protein analysis

Mouse islets or cells were solubilized in lysis buffer with protease inhibitors (Nacalai Tesque) and phosphatase inhibitors (Nacalai Tesque). The protein concentration was measured using a BCA Protein Assay Kit (Nacalai Tesque). After SDS-PAGE at 30 mA for 1 h, proteins were transferred to PVDF membrane (Millipore) using Trans-Blot Turbo (25V-1.0A; Bio-Rad) for 30 min. Immunoblotting was performed with following antibodies. Antibodies against VDAC (#4866) and UCP2 (#89326) were purchased from Cell Signaling Technology (Danvers, MA, USA). Antibodies against α-tubulin (ab7291), β-actin (ab8227), PINK1 (ab23707), HNF4α (ab201460) and total OXPHOS (ab110413) were purchased from Abcam (Cambridge, MA, USA). Antibodies against UCP2 (sc-6525) and PARKIN (sc-32282) were purchased from Santa Cruz (TX, USA). The antibodies against UCP2 (11081-1-AP), ALDOB (18065-1-AP), NRF2 (16396-1-AP) and SOD2 (24127-1-AP) were purchased from Proteintech (Rosemont, USA). Densitometry was performed using Image Lab™ software (Bio–Rad, Hercules, CA, USA). Automated quantitative western blotting was performed by Abby (ProteinSimple, San Jose, CA) according to the manufacturer’s instructions. Antibodies to the following proteins were used for the analysis of Abby: UCP2 (1:50 dilution; Proteintech, #11081-1-AP) and α-tubulin (1:50 dilution; Abcam, #ab7291). Default assay parameters were used for control and data analysis and peak areas were calculated using the Compass software (ProteinSimple).

#### qPCR and microarray analysis

Total RNA was isolated from handpicked islets, livers and hypothalamus using an RNase-free DNase and RNeasy Kit (Qiagen, Valencia, CA). The concentration of total RNA was measured by a NanoDrop One (Applied Biosystems). It was confirmed that the A260/A280 ratio was 1.8–2.1 and the A260/A230 ratio was more than 2.0 in extracted total RNA. cDNA was prepared using High Capacity cDNA Reverse Transcription Kits (Applied Biosystems) and was subjected to qPCRquantitative PCR using Sybr Green Gene Expression Assays (7900 Real-Time PCR System; Applied Biosystems) with THUNDERBIRD qPCR Master Mix (TOYOBO). The primers are listed in Table S2. A DNA microarray was performed using an Agilent-074809 Sure Print G3 Mouse GE8 × 60K Microarray (GPL21163) (Agilent, Santa Clara, CA, USA). The quality of total RNA was measured by a Bioanalyzer (Agilent), and the samples showing an RNA integrity number (RIN) of 7.0 or higher were used for microarray analysis. Data were analyzed using Genespring GX software (Agilent). The microarray data can be found at GEO: https://www.ncbi.nlm.nih.gov/geo/query/acc.cgi?acc=GSE147269.

#### Immunostaining studies

Pancreatic tissue sections from βUCP2Tg mice and in vitro-cultured human islets were immunostained with anti-insulin (Abcam), anti-UCP2 (Santa Cruz), anti-AldB (Proteintech), or anti-BrdU (Dako, Tokyo, Japan) antibodies. Images were acquired as described elsewhere (Shirakawa et al., 2013). For immunofluorescence cytochemistry (IFCC), 1 × 10^4^ MIN6-M9 cells were seeded on poly-L-lysine-coated chamber slides (Nunk, #154534PK) and infected with Ad-LacZ or Ad-Ucp2 at an MOI of 500 for 48 h. Cells were fixed with 4% PFA and were permeabilized with 0.01% Triton X-100 in PBS. Anti-HNF4α antibody was purchased from Abcam (#ab201460). Images were acquired using an FV1000-D confocal laser scanning microscope (Olympus, Japan). Super resolution STED microscopic images were acquired using TCS SP8 STED (Leica Microsystems, Germany).

#### Electron microscopy

Isolated islets were fixed in a mixture of 2% glutaraldehyde and 1% OsO4 in 0.1 mol/L phosphoric acid buffer. Islets were embedded in epoxy resin, and the blocks were trimmed with a diamond knife. Images from ultrathin sections were acquired with an HT-7500 electron microscope (Hitachi, Tokyo, Japan).

#### Mouse studies

Blood glucose and serum insulin levels were determined using Glutest Neo Super (Sanwa Chemical Co. Kanagawa, Japan) and an insulin ELISA kit (Morinaga Institute of Biological Science, Yokohama, Japan), respectively. For the oral glucose tolerance test (OGTT), all mice were denied access to food for 16–20 h before the OGTT. The insulin tolerance test (ITT) was performed by intraperitoneal injection of human insulin (0.75 mU/g body weight).

#### Islet studies

Islets were isolated from 8- to 12-week-old mice as described elsewhere (Shirakawa et al., 2011). Islets were handpicked and cultured overnight in RPMI1640 medium containing 5.6 mmol/ glucose supplemented with 10% FBS (vol./vol.) and penicillin/streptomycin (1% vol/vol). Islets were treated with 30 μmol/L glucokinase activator CpdA (GKA, Calbiochem, USA), 20 mmol/l N-acetyl-L-cysteine (Sigma–Aldrich, Switzerland), 50 μmol/L nifedipine (Sigma–Aldrich), 10 μmol/L FK506 (Sigma–Aldrich), 200 μmol/L diazoxide (Wako Pure Chemical Industries), 200 nmol/L OSI-906 (Selleck Chemicals, USA), 4 μmol/L Akt inhibitor X (Calbiochem), 10 μmol/L U0126 (Selleck Chemicals), 1 μmol/L thapsigargin (Sigma–Aldrich), 5 μmol/L genipin (Wako Pure Chemical Industries, Japan) or 30 nmol/L rapamycin (Calbiochem) for 24 h. For the study related to stimulation with insulin (Sigma–Aldrich), islets were subjected to 6 h of fasting in RPMI1640 medium containing 2.8 mmol/L glucose and 0.1% BSA (vol/vol). ATP contents in mouse islets were measured using the ADP/ATP assay kit (Abcam). Glucose-stimulated insulin secretion (GSIS) from mouse and human islets was induced as described previously (Shirakawa et al., 2011). Insulin secretion data were corrected by the insulin content of islets. One hundred nmol/l liraglutide (Novo Nordisk, Denmark), 5 μmol/L genipin (Wako Pure Chemical Industries, Japan), 10 μmol/L TAK-875 (Fasiglifam, AdooQ BioScienc, USA) or 100 nmol/L HC 067047 (Santa Cruz) were used for the mouse GSIS assay.

#### Mitochondrial respiration in islets

Mitochondrial respiration in mouse islets was measured according to a previous report (Taddeo et al., 2018). Briefly, isolated islets (20/well) from βUCP2Tg mice and WT mice or islets infected with adenoviral vectors were incubated using a culture insert for 24 h in RPMI1640 medium containing 5.6 mmol/L glucose, 1 mmol/L pyruvate (Nacalai Tesque) and 10% FBS. The islets were then washed with PBS (−) and seeded onto poly-L-lysine (Sigma)-coated XF96 cell culture microplates (Agilent Technologies) containing 160 μL/well of the assay medium. The culture microplates were then centrifuged at 500 rpm for 7 min at room temperature and incubated for 1–2 h at 37°C in a non-CO_2_ incubator. XF RPMI Medium (Agilent Technologies) containing 5.6 mmol/L glucose, 1 mmol/L pyruvate and 2 mmol/l L-glutamine (Nacalai Tesque) was used as the assay medium. The oxygen consumption rate (OCR) and extracellular acidification rate (ECAR) were measured using a Seahorse XF96 Analyzer (Agilent Technologies). Basal respiration was measured for 18 min. Then, the islets were sequentially exposed to glucose (11.1 mmol/L final concentration), oligomycin (4 μmol/L final concentration), FCCP (1 μmol/L final concentration) and rotenone/antimycin A (2.5 μmol/L final concentration) for 30 min. Wave 2.6.0 (Agilent Technologies) software was used to analyze the NonMitochondrial Oxygen Consumption, Basal Respiration, Maximal Respiration, Proton Leak, ATP Production, and Spare Respiratory Capacity.

#### Assay of mitochondrial membrane potential, mitophagy flux and oxidative stress

Mitochondrial membrane potential, mitophagy flux and oxidative stress in MIN6-M9 cells were analyzed using the MT-1 MitoMP Detection Kit (#MT13), Mitophagy Detection Kit (#MD01) and mtSOX Deep Red-Mitochondrial Superoxide Detection kit (#MT14) (Dojindo Laboratories, Kumamoto, Japan), respectively. Cells were treated according to the manufacturer’s recommendations. Images were acquired using a BZ-X800 microscope (Keyence).

#### Methylglyoxal assay in mouse islets

The methylglyoxal concentration in βUCP2Tg and WT islets was determined using a methylglyoxal colorimetric assay kit (#K500-100, BioVision) according to the manufacturer’s instructions. One hundred islets were lysed in 50 μL of 0.1% Triton X- in PBS, and 20 μL was used to measure the methylglyoxal concentration.

#### Intracellular Ca^2+^ measurement

For the mouse islet study, islets were incubated with 5 μmol/L Fluo-8 (AAT Bioquest) for 40 min and then transferred to poly-L-lysine-coated glass-bottom dishes containing Hanks’ balanced salt solution (HBSS). For the fasiglifam stimulation experiment, Ca^2+−^free HBSS containing 2.8 mmol/L glucose supplemented with 0.1 mmol/L EGTA, 200 μmol/L diazoxide and 50 μmol/L nifedipine was used. Images were acquired using a BZ-X800 microscope (Keyence). For INS1 cells, 2.5 × 10^4^ cells were seeded on 96-well plates and infected with Ad-LacZ + Sh-Scramble, Ad-Ucp2 + Sh-Scramble or Ad-Ucp2 + Sh-AldB at an MOI of 10 in the presence of 11.1 mmol/L glucose for 48 h. INS1 cells were incubated in HBSS containing 2.8 mmol/L glucose and 5 μmol/L Fluo-8 and then washed twice with HBSS. The fluo-8 intensity in the cells was measured every 5 s for 180 s by using an EnSpire Multimode Plate Reader (Parkin Elmer). Cells were stimulated with 25 mmol/L glucose HBSS at 30 s. To measure ER Ca^2+^ in INS1 cells, we used the D1ER plasmid (Palmer et al., 2004). pcDNA-D1ER was a gift from Amy Palmer & Roger Tsien (Addgene plasmid # 36325; http://n2t.net/addgene:36325; RRID:Addgene_36325). A total of 2.5 × 10^4^ INS1 cells were seeded on 96-well plates and infected with Ad-LacZ or Ad-Ucp2 at an MOI of 10 in the presence of 11.1 mmol/L glucose for 48 h. D1ER plasmid (0.1 μg) was transfected into cells by using METAFECTENE PRO (Biontex, Germany) 24 h before the Ca^2+^ assay. Two hours prior to measurement, the medium was changed to HBSS containing 2.8 mmol/L glucose. The intensity of the D1ER was measured every 5 s by an EnSpire Multimode Plate Reader (Parkin Elmer). Then, 25 mmol/L glucose was added at 30 s, and 10 μmol/L fasiglifam was added at 180 s. Fluorescence measurements were made using the following wavelengths: Fluo-8 excitation at 490 nm with emission at 525 nm, D1ER excitation at 440 nm with emission at 485 nm.

### Quantification and statistical analysis

All the data are expressed as the means ± SEM One-way ANOVA with post hoc analysis was used to compare the values among the different experimental groups. When only two groups were compared, a two-tailed Student’s *t* test was used. For ANOVA, the homogeneity of variance was tested by Levene’s test. If the results were similar, Tukey’s test or Bonferroni’s test was performed, and if not, Dunnett’s test was adopted. For the two-tailed Student’s *t* test, variances between two groups were compared by the F test. Prism 8 (GraphPad Software, San Diego, CA, USA) and SPSS software (IBM, Armonk, NY, USA) were used for all statistical analyses. Differences were considered significant if the p value was <0.05 (∗) or <0.01 (∗∗).

## Data Availability

Microarray data for βUCP2Tg islets have been deposited at GEO. Accession number is GSE147269. The microarray data can be found at GEO: https://www.ncbi.nlm.nih.gov/geo/query/acc.cgi?acc=GSE147269. This paper does not report original code.
