# Supplementary material for: Uncoupling protein 2 and aldolase B impact insulin release by modulating mitochondrial function and Ca2+ release from the ER
Source: iScience. 2022 Jun 14;25(7):104603. doi: 10.1016/j.isci.2022.104603 (PMC9253497; doi:10.1016/j.isci.2022.104603)

iScience, Volume *25*

Supplemental information

Uncoupling protein 2 and aldolase B impact insulin release by modulating mitochondrial function and Ca^{2+} release from the ER

Ryota Inoue, Takahiro Tsuno, Yu Togashi, Tomoko Okuyama, Aoi Sato, Kuniyuki Nishiyama, Mayu Kyohara, Jinghe Li, Setsuko Fukushima, Tatsuya Kin, Daisuke Miyashita, Yusuke Shiba, Yoshitoshi Atobe, Hiroshi Kiyonari, Kana Bando, A.M. James Shapiro, Kengo Funakoshi, Rohit N. Kulkarni, Yasuo Terauchi, and Jun Shirakawa

Supplemental information

Supplementary Figures

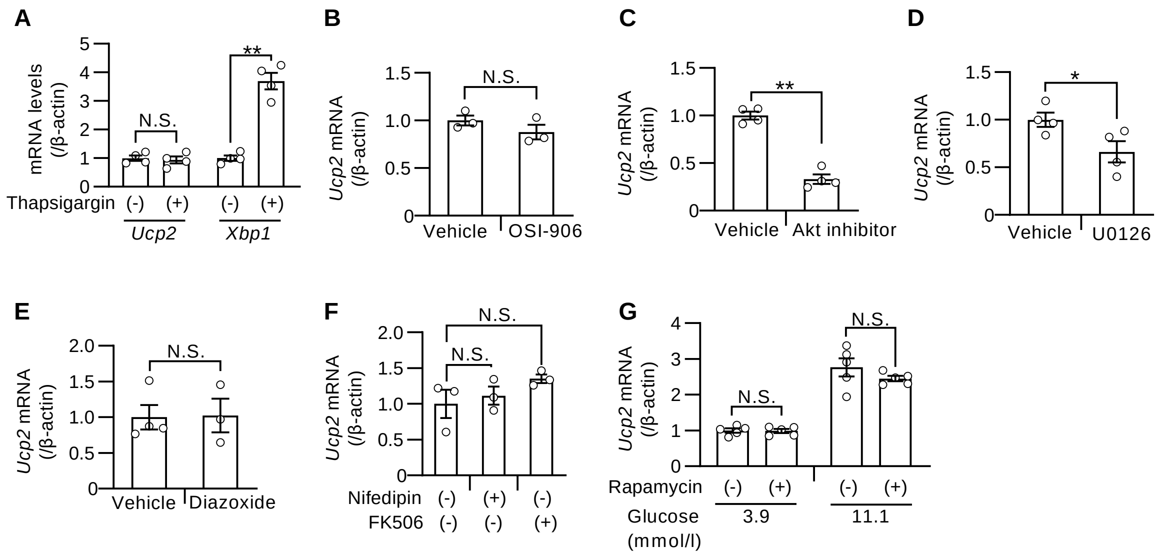

Figure S1. The regulation of UCP2 expression in mouse islets (related to Figure 1).
(A-G) *Ucp2* mRNA levels in islets from C57BL/6J mice. Islets were treated with the following drugs for 24 hours.
(A) 1 µmol/l thapsigargin. The graphs also show the mRNA level of *Xbp1*, an ER stress marker (n = 4 per group).
(B) 200 nmol/l OSI-906 (n = 3 per group).
(C) 4 µmol/l Akt inhibitor X (n = 4 per group).
(D) 10 µmol/l U0126 (n = 4 per group).
(E) 200 µmol/l diazoxide (Vehicle: n = 4, diazoxide: n = 3).
(F) 50 µmol/l nifedipine or 10 µmol/l FK-506 (n = 3 per group).
(G) Rapamycin (30 nmol/l) in the presence of 3.9 or 11.1 mmol/l glucose (n = 5 per group).
Data are the means ± SEM. *p < 0.05, **p < 0.01. N.S.: not significant. The two-tailed Student's t test was used in A-E. One-way ANOVA was used in F and G.

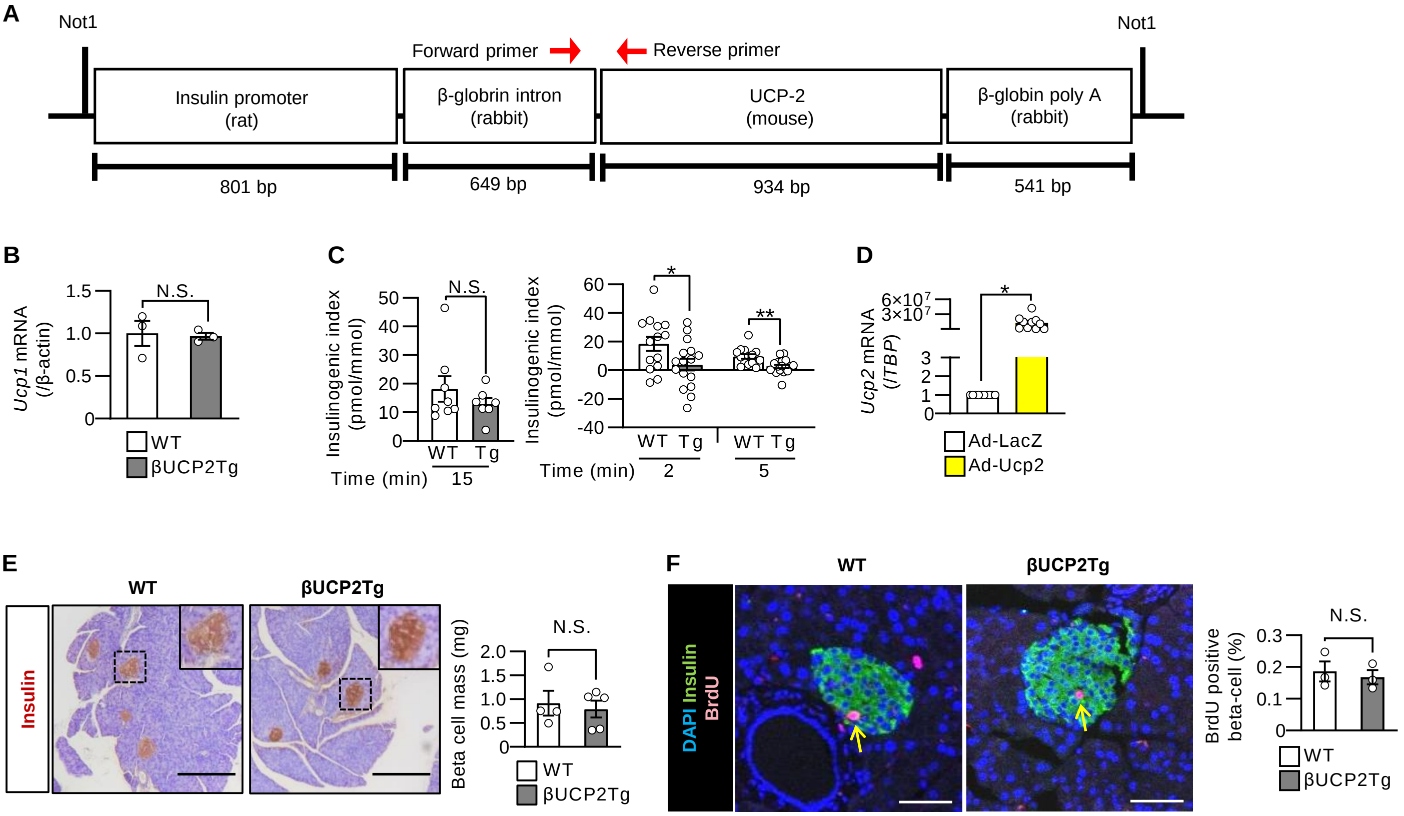

Figure S2. Phenotype of βUCP2Tg mice (related to Figure 2).

(A) Gene construct of βUCP2Tg mice. The red arrow shows the genotyping primers.

(B) *Ucp1* mRNA levels in βUCP2Tg and WT islets. Islets were incubated in the presence of 11.1 mmol/l glucose for 24 hours (n = 3 per group).

(C) Insulinogenic index in βUCP2Tg and WT mice at 15 minutes (left panel, WT: n = 7, βUCP2Tg: n = 8, 1.5 g/kg BW glucose) and at 2 and 5 min (right panel, WT: n = 14, βUCP2T: n = 17, 2.5 g/kg BW glucose) after glucose loading. The insulinogenic index was calculated as follows: [(serum insulin level at 2, 5 or 15 min) – (serum insulin level at 0 min)]/[(blood glucose level at 2, 5 or 15 min) – (blood glucose level at 0 min)].

(D) *Ucp2* mRNA levels in human islets infected with Ad-LacZ or Ad-Ucp2 at 3 × 10^6 MOI in the presence of 5.6 mmol/l glucose for 48 hours (n = 10 per group).

(E) β-cell mass in βUCP2Tg and WT mice (WT: n = 4, βUCP2Tg: n = 5). Representative images of the pancreas show brown staining of insulin. The scale bar represents 100 μm.

(F) BrdU incorporation in βUCP2Tg and WT islets. Insulin is stained green, nuclei are stained blue with DAPI, and BrdU-positive nuclei are stained pink. The yellow arrows indicate BrdU-positive β-cells. The scale bar represents 50 μm. The ratio of BrdU-positive β-cells to the total count of insulin-positive β-cells is shown (n = 3 per group).

Data are the means ± SEM. N.S.: not significant. The two-tailed Student’s t test was used in B-F.

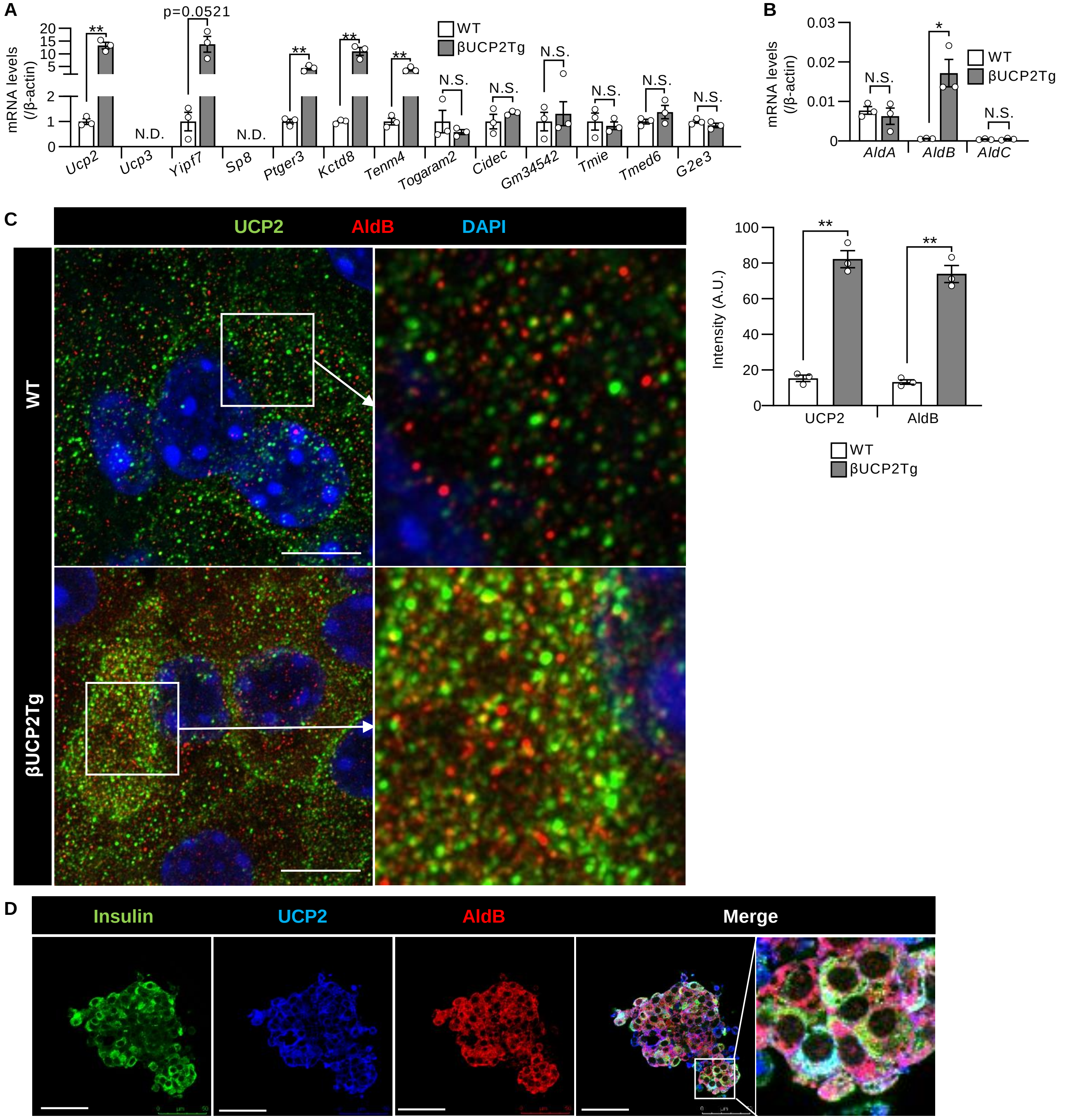

Figure S3. Results of qPCR and immunostaining in mouse and human islets (related to Figure 4).

(A) Quantitative PCR data for βUCP2Tg and WT islets. Genes were significantly increased or decreased in islets from βUCP2Tg mice in the microarray analysis. Islets were incubated in the presence of 11.1 mmol/l glucose for 8 hours (n = 3 per group). N.D.: not detected.

(B) mRNA levels of *AldA*, *AldB* and *AldC* in βUCP2Tg and WT islets. Islets were incubated in the presence of 11.1 mmol/l glucose for 24 hours (n = 3 per group).

(C) Immunostaining of pancreases from βUCP2Tg and WT mice. Images were acquired using stimulated emission depletion (STED) microscopy. The intensities of UCP2 (green) and AldB (red) (n = 3 per group) were calculated using ImageJ. The scale bar indicates 5 μm.

(D) Immunostaining of human islets. Insulin is stained green, UCP2 is stained blue and AldB is stained red. Human islets were incubated with 11.1 mmol/l glucose. The scale bar represents 50 μm.

Data are the means ± SEM. *$p < 0.05$, **$p < 0.01$. N.S.: not significant. The two-tailed Student's t test was used in A-C.

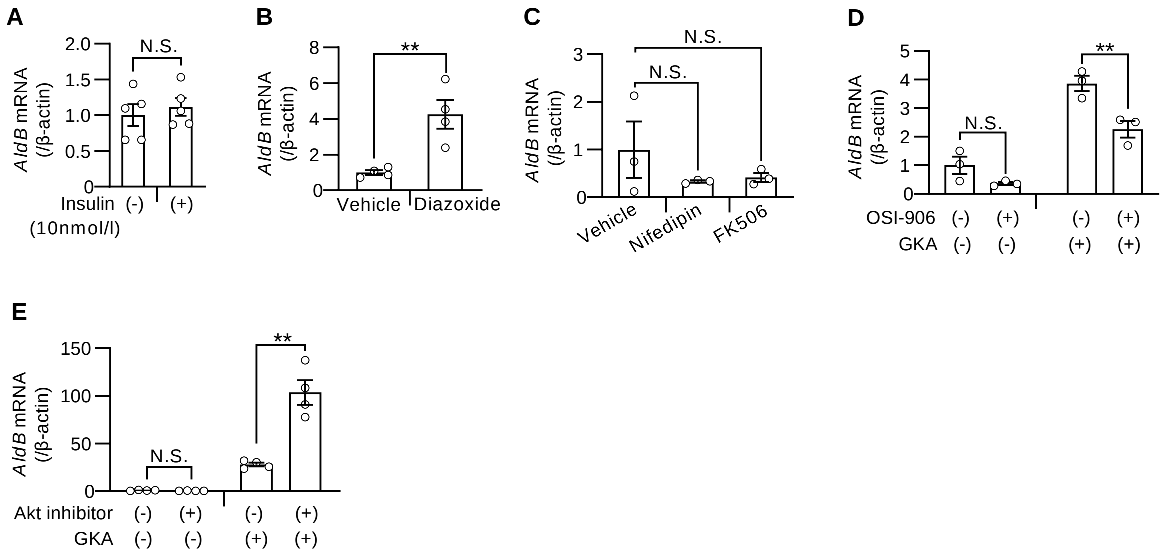

Figure S4. Regulation of AldB expression in mouse islets (related to Figure 5).
(A-E) *AldB* mRNA levels in islets of C57BL/6J mice. Islets were treated with the following drugs for 12 hours (A) or 24 hours (B-E).
(A) 10 nmol/l insulin in the presence of 2.8 mmol/l glucose (n = 5 per group).
(B) 200 µmol/l diazoxide in the presence of 5.6 mmol/l glucose (n = 4 per group).
(C) 50 µmol/l nifedipin or 50 µmol/l FK506 in the presence of 5.6 mmol/l glucose (n = 3 per group).
(D) OSI-906 (200 nmol/l) in the presence or absence of 30 µmol/l GKA (n = 3 per group). Glucose concentration in culture medium was 5.6 mmol/l.
(E) Akt inhibitor X (4 µmol/l) in the presence or absence of 30 µmol/l GKA (n = 4 per group). Glucose concentration in culture medium was 5.6 mmol/l.
Data are the means ± SEM. **p < 0.05, **p < 0.01. N.S.: not significant. The two-tailed Student’s t test was used in A and B. One-way ANOVA was used in C-E.

Supplementary Tables

Table S1. Characteristics of human islet donors, related to STAR Methods.

ID#	Age (years)	Sex	BMI	Diabetic donor status	Purity (%)	Viability (%)	Related to
1	45	Female	29.7	No	80	N/A	Figure S3D
2	65	Female	20.8	No	90	N/A	Figures 2J
3	31	Male	31.9	No	85	N/A	Figures 2J and S2D
4	58	Male	27.8	No	75	N/A	Figures 2J, 5C and S2D
5	49	Female	27.2	No	72.5	85.5	Figures 2J and S2D
6	55	Female	26.0	No	42.5	89.5	Figures 2J and S2D
7	24	Male	24.1	No	27.5	74	Figure S2D
8	36	Male	23.1	No	35	98	Figure S2D
9	55	Male	24.3	No	30	82	Figure S2D
10	69	Female	30.1	No	40	85	Figure S2D
11	61	Male	39.5	No	35	96	Figure S2D
12	73	Female	34.3	No	55	N/A	Figure S2D

Table S2. qPCR primer sequences, related to STAR Methods.

Species	Gene	Sequence (Forward)	Sequence (Reverse)
Mouse	β-actin	GGCTGTATTCCCCTCCATCG	CCAGTTGGTAACAATGCCATGT
Mouse	Ucp1	AGGCTTCCAGTACCATTAGGT	CTGAGTGAGGCAAAGCTGATTT
Mouse	Ucp2	TTGGCGGTATCCAGAGGGAA	ATGGTTGGTTTCAAGGCCACA
Mouse	Ucp3	TACCCAACCTTGGCTAGACG	GTCCGAGGAGAGAGCTTGC
Mouse	Irs-2	CTGCGTCCTCTCCCAAAGTG	GGGGTCATGGGCATGTAGC
Mouse	Drp1	TAAGCCCTGAGCCAATCCATC	CATTCCCGGTAAATCCACAAGT
Mouse	Opa1	TGGAAAATGGTTCGAGAGTCAG	CATTCCGTCTCTAGGTTAAAGCG
Mouse	Mfn1	CCTACTGCTCCTTCTAACCCA	AGGGACGCCAATCCTGTGA
Mouse	Tfam	GAGGCAAAGGATGATTCGGCTC	CGAATCCTATCATCTTTAGCAAGC
Mouse	Aldoa	CGCGTTCGCTCCTTAGTCC	GACAGGCGGGTCATGTTGAA
Mouse	Aldob	AATGGGCTGGTCCCTATTGTT	GGCAGTGCTCCAGGTCATG
Mouse	Aldoc	CTGGGAAGCCTGTTTGGTTAG	TGAGTGGGGCATGATGACAG
Mouse	Yipf7	GCCTTGGGATTCACGCCTTA	ATGGGGAGCAAGCAGTATCC
Mouse	Sp8	GCGCACACTTGCACCATATC	TTCTTCTCGCGTTCCCCTTC
Mouse	Ptger3	ATCATGTGTGTGCTGTCCGT	TCAGGTTGTTCATCATCTGGCA
Mouse	Kctd8	TGTTTTCCAGAGCGCAAACG	ATTCACACCGGCCATCAGTT
Mouse	Tenm4	TCTGGACTCCTCCACTTGACA	TACGTCCCAGGGGTGAGTAG
Mouse	Togaram2	GTCATGAGGGTGTGGAGACG	GATGGGAAGCATCTGTGCGA
Mouse	Cidec	GGGAGGTCCAACACAATCCAA	CTCCAAGCTGTGAGCCATGA
Mouse	Gm34542	GAAGGGAGATGGGCTAGTGC	CATGCCAGGGACTAAAGGGG
Mouse	Time	GCCTTTTACCTCCCTCTCCAG	GGTGGGTAAGGAAATCGGGG
Mouse	Tmed6	CCCCGCAGGGTTTCCTTATT	TCGCATCCAGAGTGTCGTTC
Mouse	G2e3	CCCCAAACACCCATTAGCCA	TTTGCTGCCTTGCCTCAGTA
Mouse	Hnf4a	CGTCCCTCGGCACTGTCC	TCCTCCAGGCTCACTTGC
Mouse	Trpv1	CCGGCTTTTTGGGAAGGGT	GAGACAGGTAGGTCCATCCAC
Mouse	Trpv2	GGACCCAAATCGGTTTGACC	GCGCAGGTACTCTAGCAGTC
Mouse	Trpv3	ACGGTCACCAAGACCTCTC	GACTGTTGGGATTGGATGGGG
Mouse	Trpv4	AAACCTGCGTATGAAGTTCCAG	CCGTAGTCGAACAAGGAATCCA
Mouse	Tbp	GGGGAGCTGTGATGTGAAGT	CCAGGAAATAATTCTGGCTCA
Mouse	Nrf2	CGAGATATACGCAGGAGAGGTAAGA	GCTCGACAATGTTCTCCAGCTT
Mouse	Sod2	CAGACCTGCCTTACGACTATGG	CTCGGTGGCGTTGAGATTGTT
Mouse	Ddit3	CTGGAAGCCTGGTATGAGGAT	CAGGGTCAAGAGTAGTGAAGGT
Mouse	Atf4	CCTGAACAGCGAAGTGTTGG	TGGAGAACCCATGAGGTTTCAA
Mouse	Atf6	TCGCCTTTTAGTCCGGTTCTT	GGCTCCATAGGTCTGACTCC
Mouse	Ern1	ACACCGACCACCGTATCTCA	CTCAGGATAATGGTAGCCATGTC
Mouse	Itpr1	CTCTGTATGCGGAGGGATCTAC	GCGGAGTATCGATTCATAGGAC
Mouse	Carl	GACTGGGATGAACGAGCCAA	GGTTTCCACTCGCCCTTGTA
Mouse	Atp2a2	TCTGGCCACTCATGACAACC	ATCATGATGACCCGGATGCC
Mouse	Crif1	GAACGCTGGGAGAAAATTCA	ATAGTTCCTGGAAGCGAGCA
Mouse	Ndufb8	GGCCGCCAAGAAGTATAACA	TGATACCACGGATCCCTCTC
Human	TBP	GCCAGCTTCGGAGAGTTCTGGGATT	CGGGCACGAAGTGCAATGGTCTTTA

Data S1. Uncropped images of immunoblots, related to Figure 1-8.

Figure 1H

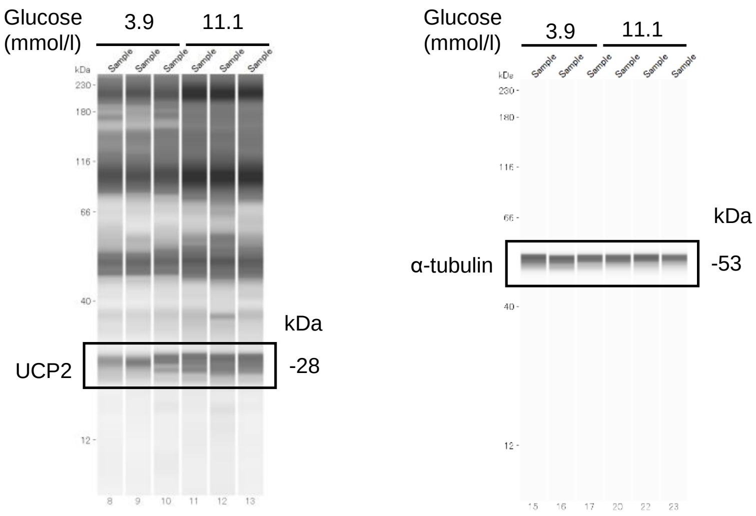

Figure 1I

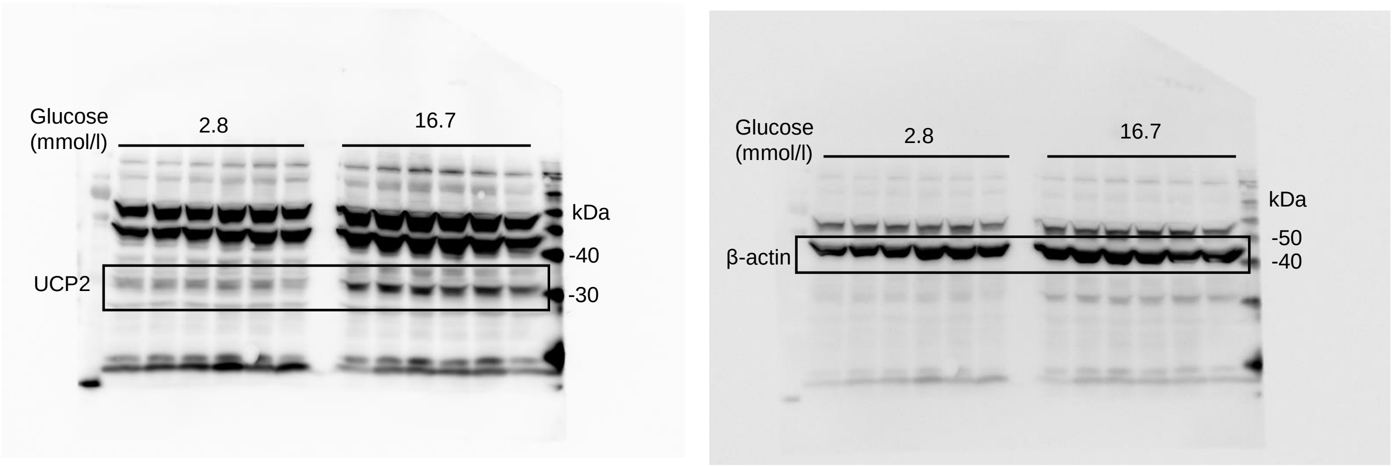

Figure 2E

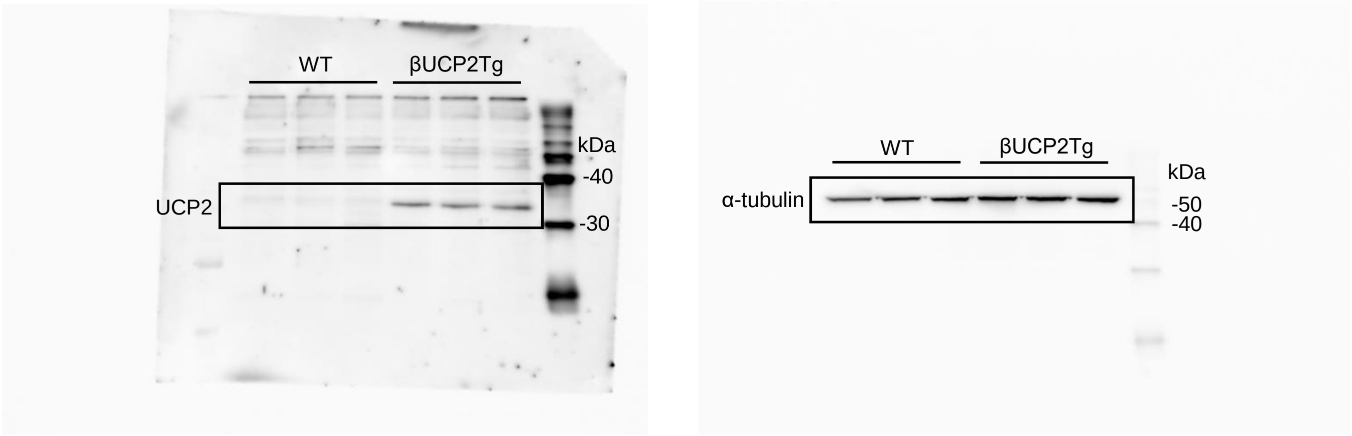

Figure 2H

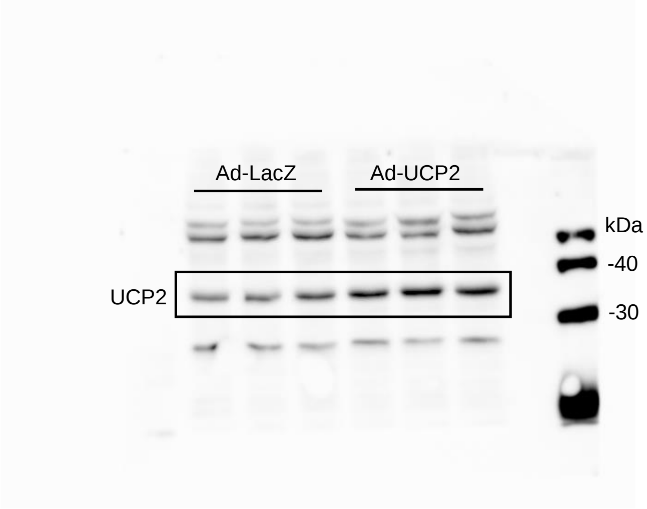

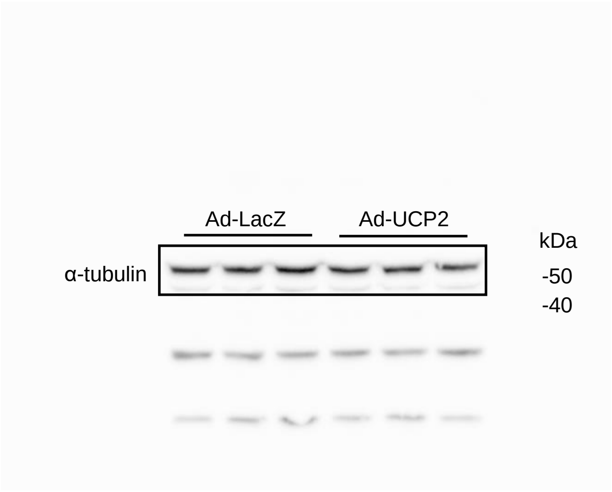

Figure 3E

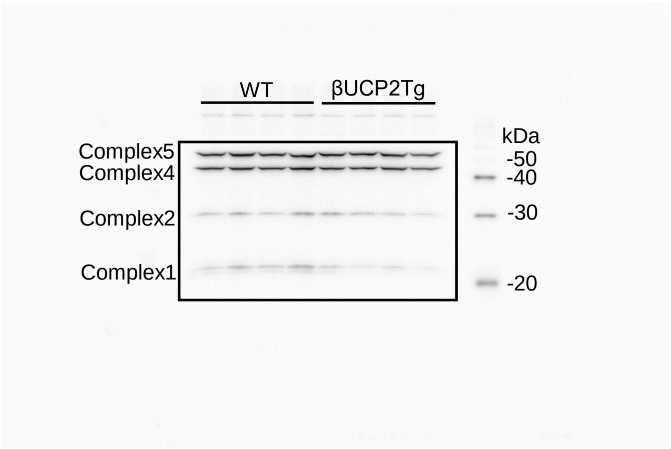

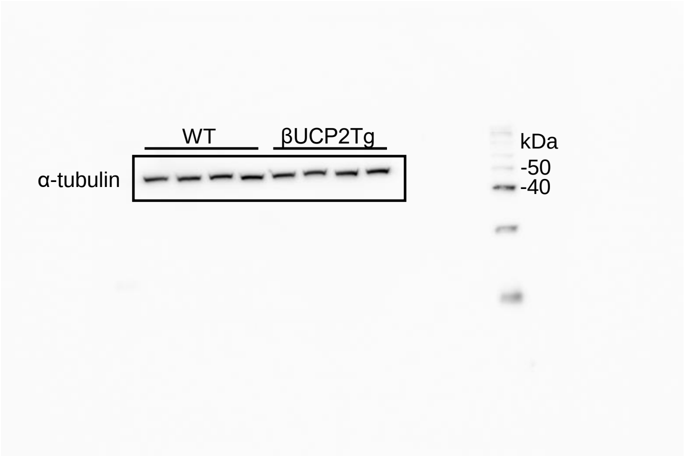

Figure 3H

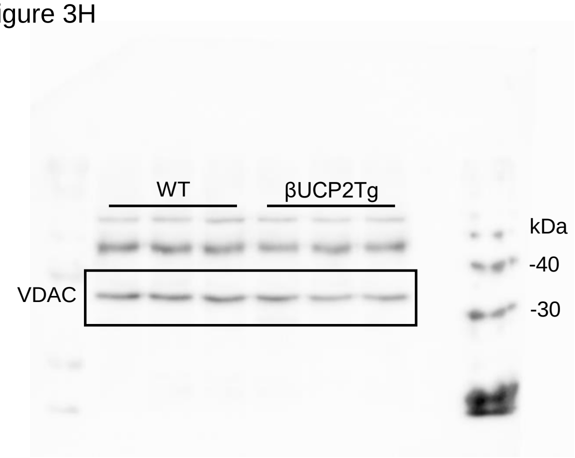

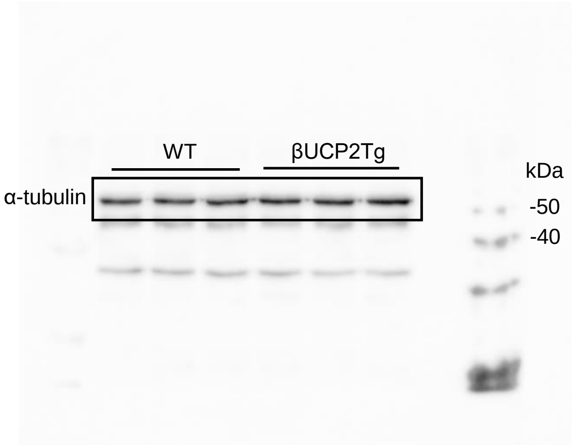

Figure 3H

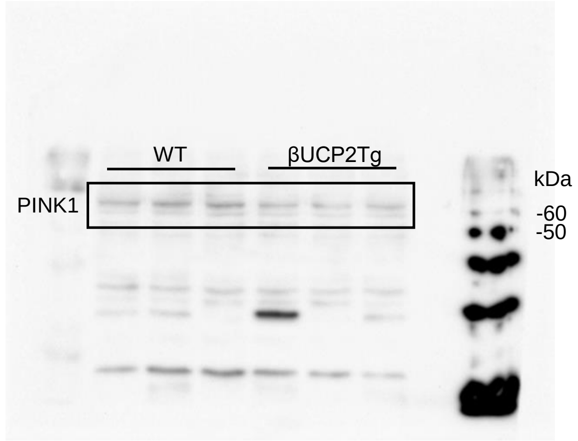

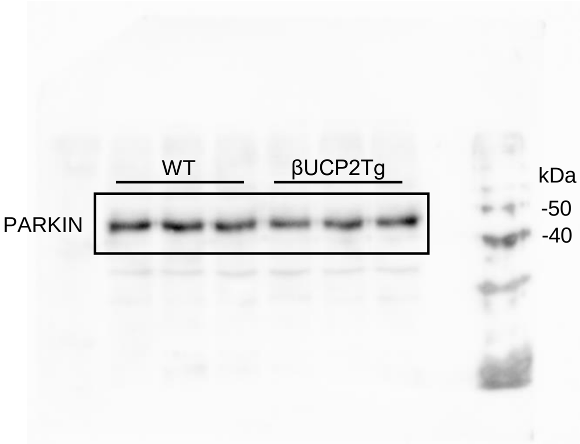

Figure 4E

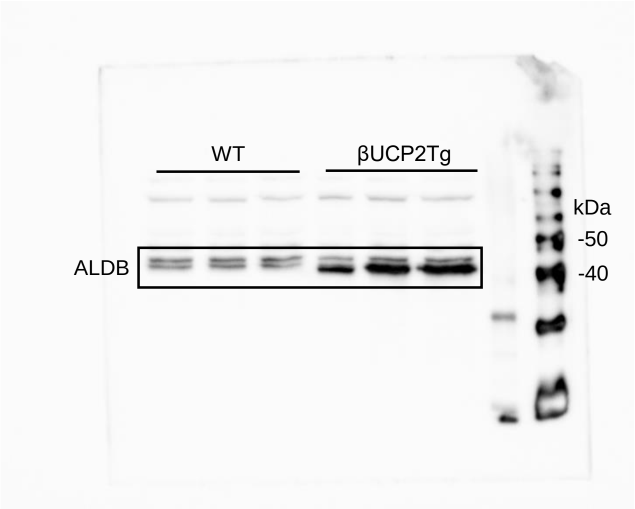

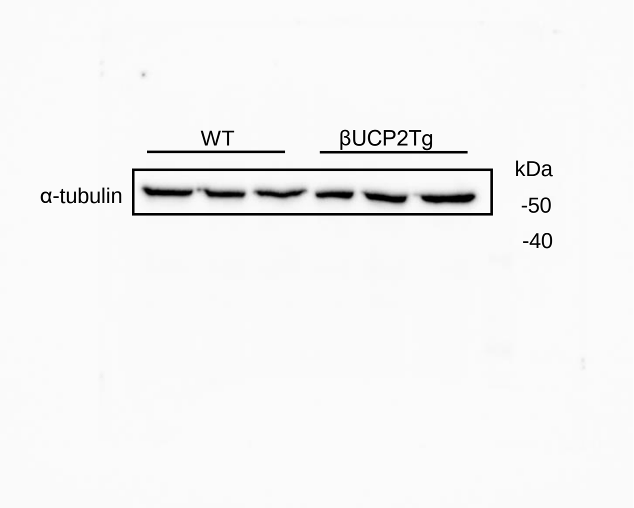

Figure 5A

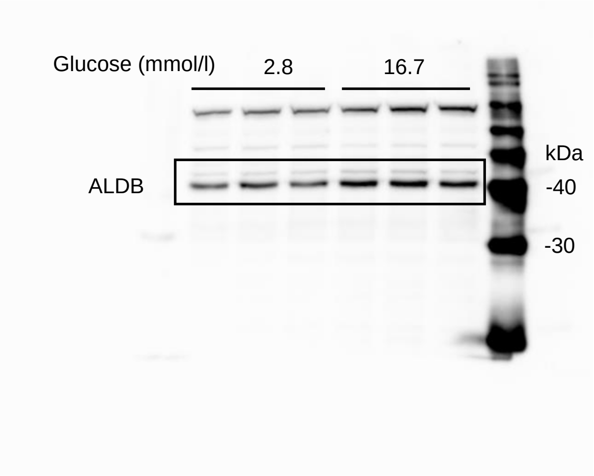

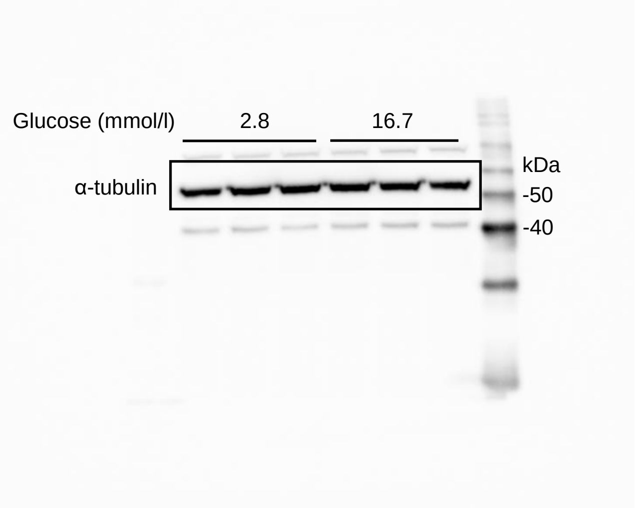

Figure 5B

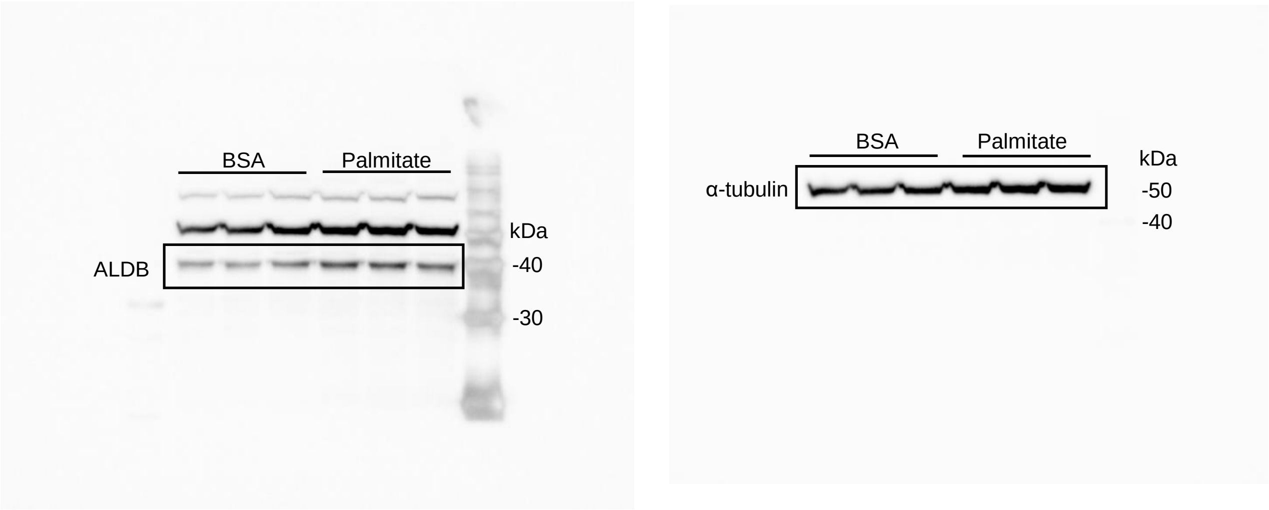

Figure 5J

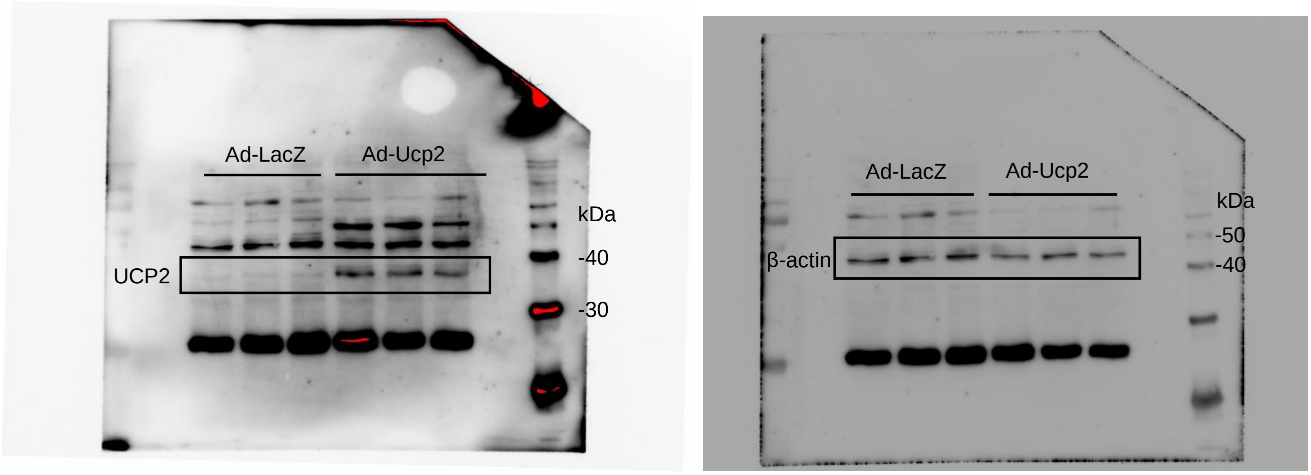

Figure 6F

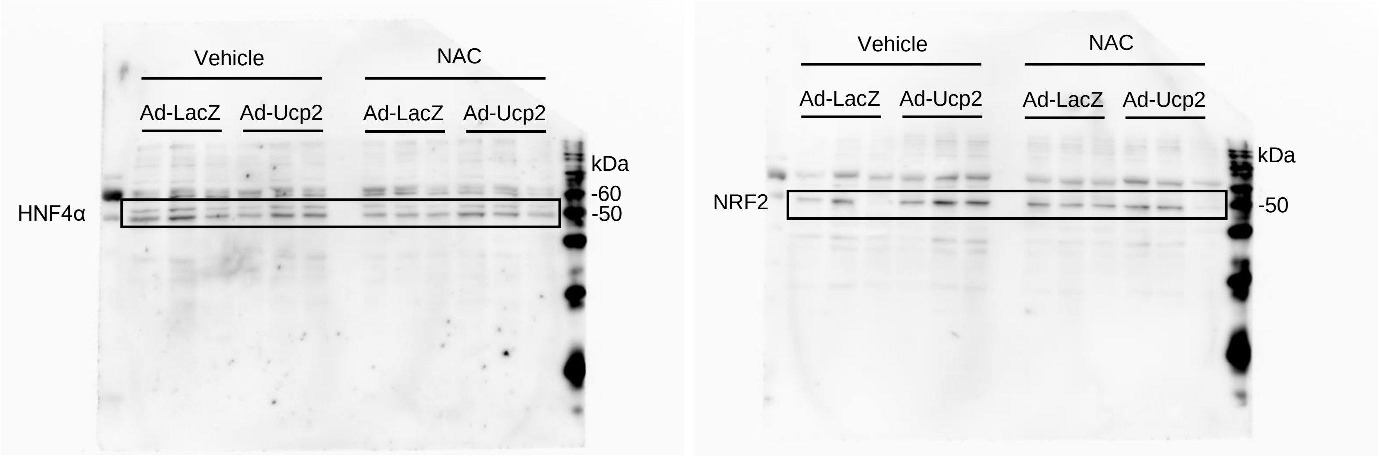

Figure 6F

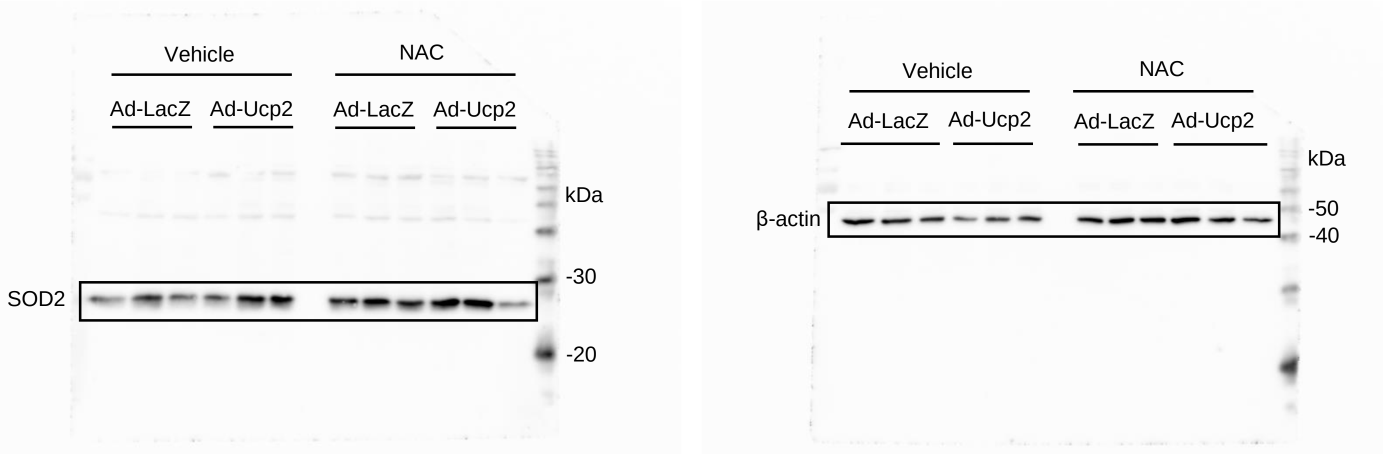

Figure 6J

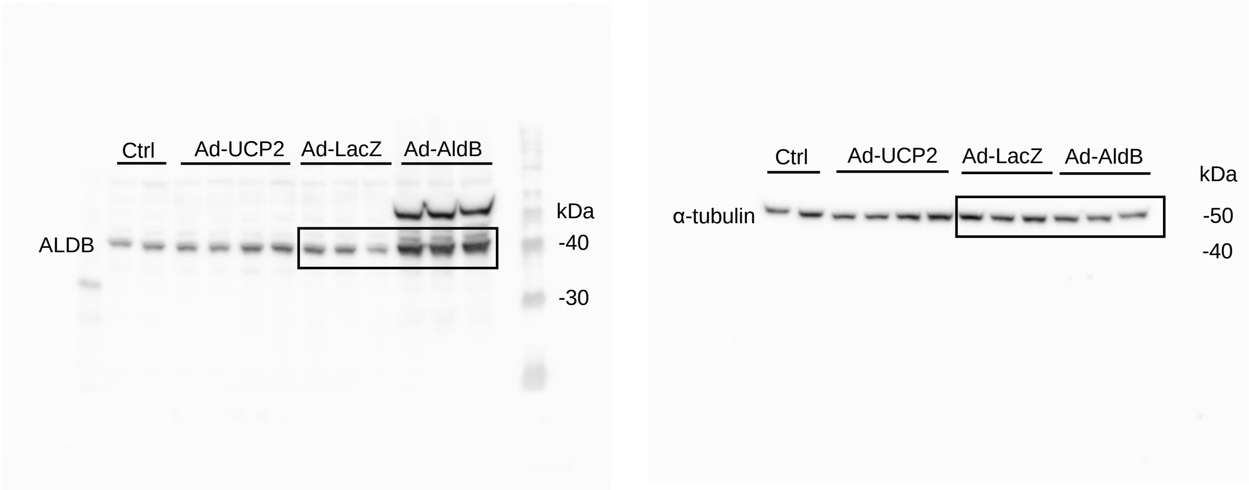

Figure 7B

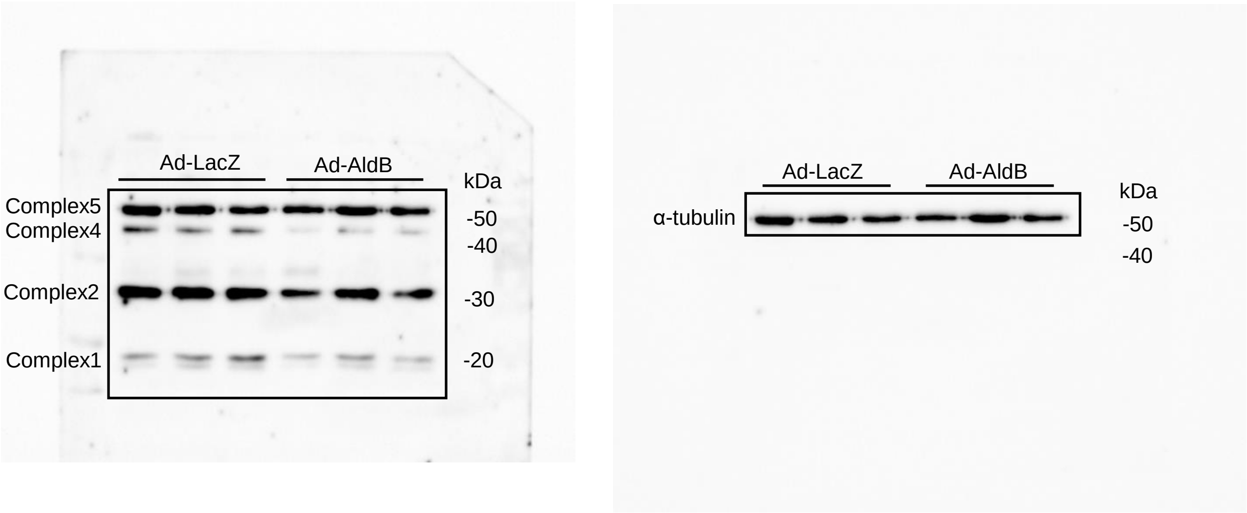

Figure 7C

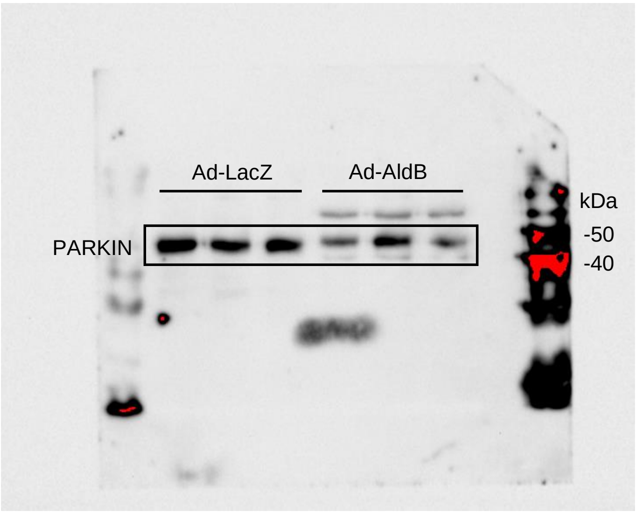

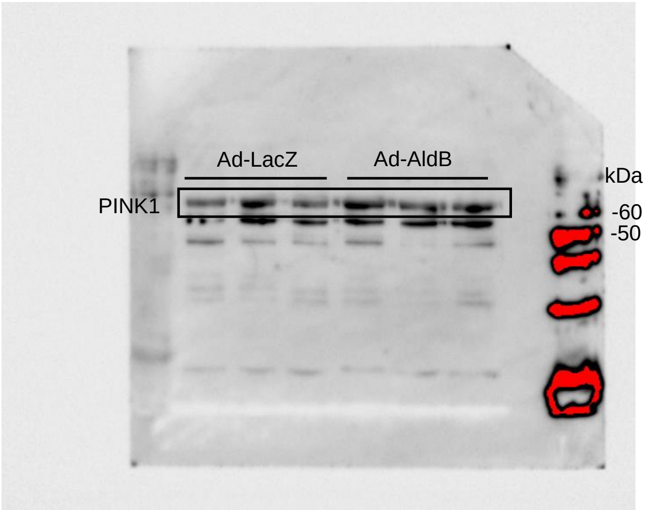

Figure 7C

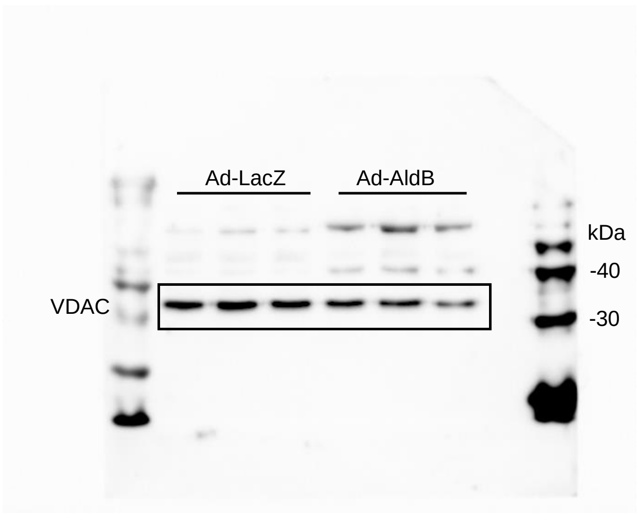

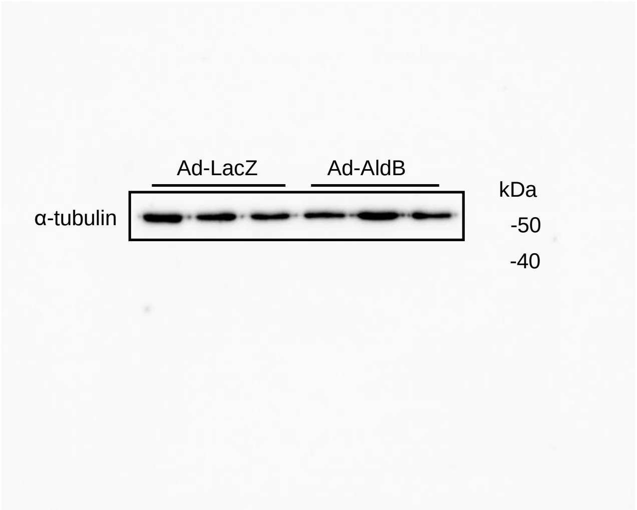

Figure 8R

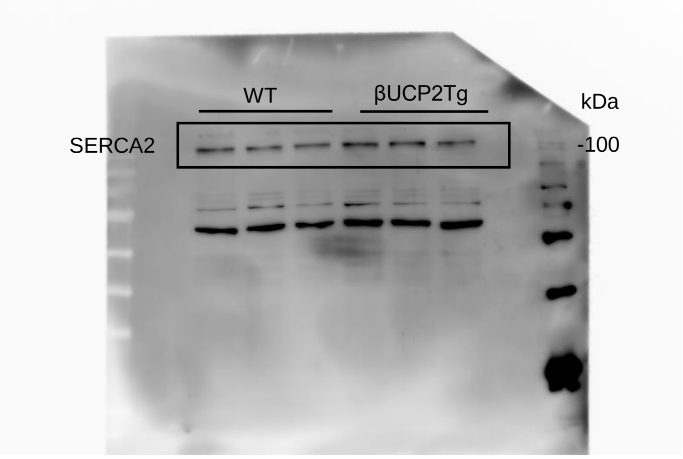

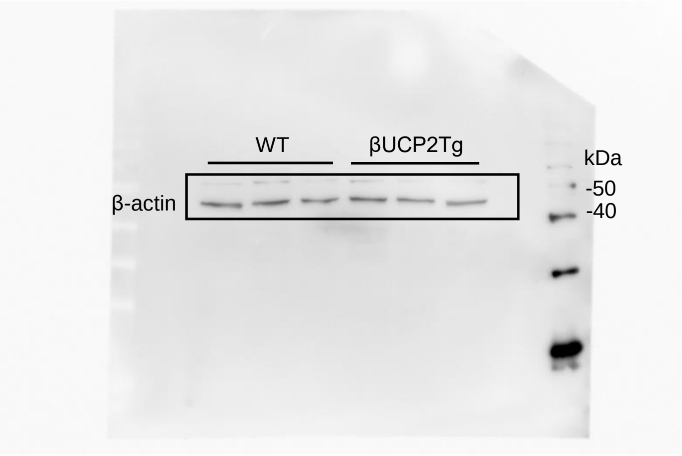

Supplement: Document S1. Figures S1–S4, Data S1, Tables S1 and S2 [file mmc1.pdf]
